# Complete mitochondrial genome of *Belligobio pengxianensis* (Cypriniformes: Gobionidae)

**DOI:** 10.1080/23802359.2023.2192310

**Published:** 2023-03-25

**Authors:** Bo Xuan, Mingyue Li, Xiaomin Ni, Cuizhang Fu

**Affiliations:** Ministry of Education Key Laboratory for Biodiversity Science and Ecological Engineering, Coastal Ecosystems Research Station of the Yangtze River Estuary, Institute of Biodiversity Science and Institute of Eco-Chongming, School of Life Sciences, Fudan University, Shanghai, China

**Keywords:** Cypriniformes, Gobionidae, upper Yangtze River, *Hemibarbus*, *Belligobio pengxianensis*

## Abstract

*Belligobio pengxianensis* is a small fish endemic to the upper Yangtze River of China. In this study, the complete mitochondrial genome of *B. pengxianensis* is determined for the first time, and it should become a reference sequence to aid in species identification, biodiversity monitoring and conservation. The mitogenome has overall length of 16,610 bp and AT content of 55.23%, including 13 protein-coding genes, two ribosomal RNAs, 22 transfer RNAs, and one non-coding control region. The results of phylogenetic analyses show that *B. pengxianensis* is nested within the genus *Hemibarbus*.

## 1. Introduction

Fishes of the family Gobionidae (Teleostei: Cypriniformes) include about 28 genera and 226 species widely distributed in drainages across East Asia and Europe (Fricke et al. 2022). The gobionid fishes can be divided into three major clades—the *Hemibarbus*-*Squalidus* clade, Sarcocheilichthyinae, and Gobioninae—where the *Hemibarbus*-*Squalidus* clade is composed of the three genera *Belligobio, Hemibarbus,* and *Squalidus* (Tan and Armbruster 2018). *Belligobio* is comprised of three species: *B. eristigma* Jordan & Hubbs 1925, *B. nummifer* (Boulenger, 1901), and *B. pengxianensis* Lu, Luo & Chen 1977 (Bănărescu 1992; Yue 1998).

**Figure 1.**
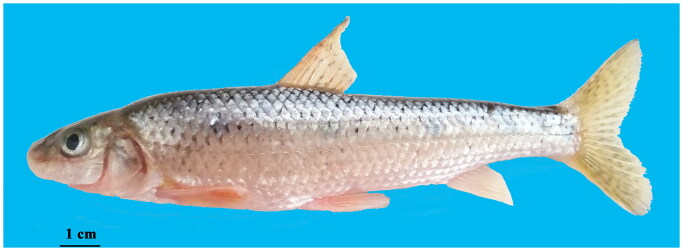
Female *B. pengxianensis* from Tongji Town, Pengzhou City, Sichuan Province, China. Photographed by Cuizhang Fu on September 12, 2015.

*B. pengxianensis* (Figure 1) is a small (standard length of less than 13 cm) fish (Yue 1998; Li 2020). In the very few studies (Ding 1994; Wang et al. 2017), it was considered to be rare, on the basis of its known records only in the Jianjiang River (a tributary of the upper Yangtze River). In a recent study (Li 2020), *B. pengxianensis* has been found to also be distributed in the Fujiang River (a tributary of the upper Yangtze River) based on molecular species delimitation using the mitochondrial cytochrome *b* gene and eight nuclear loci. Li (2020) has also indicated that *B. pengxianensis* and *B. nummifer* could not be differentiated by previously defined diagnostic characteristics (Yue 1998), such that prior records on *B. nummifer* in multiple tributaries of the upper Yangtze River (Ding 1994; Lei et al. 2015; Chen et al. 2017) might be due to misidentification of *B. pengxianensis*. Therefore, the ambiguities in species identification between *B. pengxianensis* and *B. nummifer*, based on morphological characteristics, require molecular evidence to delimit species boundaries.

In this study, the complete mitochondrial genome of *B. pengxianensis* is determined for the first time. The mitogenome should become a reference sequence to aid in molecular species identification, biodiversity monitoring and conservation, and is also useful in clarifying phylogenetic position of *B. pengxianensis*.

## 2. Materials

The specimen of *B. pengxianensis* was collected in a dead state from Tongji Town, Pengzhou City, Sichuan Province, China (31.1611°N, 103.8394°E) on 12 September 2015. We obtained the sample from the fish catches of a local fisherman. It was placed in the Zoological Museum of Fudan University under the voucher number FDZM-BPPZ20150912-01 (Cuizhang Fu, czfu@fudan.edu.cn).

## 3. Methods

Total genomic DNA was extracted from muscle tissues using a high-salt method (Miller et al. 1988). Thirteen primer pairs were used to amplify DNA fragments, with the same PCR system and settings as Chai and Fu (2020). The forward primer of Primer4, reverse primer of Primer6 as well as primers of Primer12 were newly designed in this study (supplemental Table S1 in Appendix I), while the remaining primers have been previously published (Chai and Fu, 2020). A total of 13 segments were obtained through Sanger sequencing using the ABI 3730 platform (Applied Biosystems, Foster City, USA). Using a reference genome from *B. nummifer* (KJ413052; Wang et al. 2016), contiguous and overlapping sequences were assembled into mitochondrial genome by Sequencher 5.4 (Gene Codes, Ann Arbor, MI), based on the default parameters. Then, the assembled mitogenome was annotated by MitoAnnotator (Iwasaki et al. 2013; http://mitofish.aori.u-tokyo.ac.jp/annotation/input.html). The genome map was drawn using the CGView Server (Grant and Stothard 2008).

**Table 1.** Characteristics of *B. pengxianensis* mitogenome.

Element	From	To	Length (bp)	Start codon	Stop codon	Strand*
*tRNA^Phe^*	1	69	69			H
*12S rRNA*	70	1028	959			H
*tRNA^Val^*	1029	1100	72			H
*16S rRNA*	1101	2792	1692			H
*tRNA^Leu^*	2793	2868	76			H
*ND1*	2870	3844	975	ATG	TAA	H
*tRNA^Ile^*	3849	3920	72			H
*tRNA^Gln^*	3919	3989	71			L
*tRNA^Met^*	3991	4059	69			H
*ND2*	4060	5104	1045	ATG	T--	H
*tRNA^Trp^*	5105	5175	71			H
*tRNA^Ala^*	5178	5246	69			L
*tRNA^Asn^*	5248	5320	73			L
*tRNA^Cys^*	5352	5419	68			L
*tRNA^Tyr^*	5421	5491	71			L
*COX1*	5493	7043	1551	GTG	TAA	H
*tRNA^Ser^*	7045	7113	71			L
*tRNA^Asp^*	7118	7189	72			H
*COX2*	7203	7893	691	ATG	T--	H
*tRNA^Lys^*	7894	7969	76			H
*ATPase8*	7971	8135	165	ATG	TAA	H
*ATPase6*	8129	8812	684	ATG	TAA	H
*COX3*	8812	9595	784	ATG	T--	H
*tRNA^Gly^*	9596	9668	73			H
*ND3*	9669	10017	349	ATG	T--	H
*tRNA^Arg^*	10018	10087	70			H
*ND4L*	10088	10384	297	ATG	TAA	H
*ND4*	10378	11760	1382	ATG	TA-	H
*tRNA^His^*	11760	11828	69			H
*tRNA^Ser^*	11829	11897	69			H
*tRNA^Leu^*	11899	11971	73			H
*ND5*	11972	13807	1836	ATG	TAG	H
*ND6*	13804	14325	522	ATG	TAA	L
*tRNA^Glu^*	14326	14394	69			L
*Cytb*	14399	15539	1141	ATG	T--	H
*tRNA^Thr^*	15540	15611	72			H
*tRNA^Pro^*	15611	15680	70			L
*D-loop*	15681	16610	930			H

*H: gene is transcribed from the heavy strand; L: gene is transcribed from the light strand.

Bănărescu (1992) has treated *Belligobio* as a sub-genus of *Hemibarbus*, while Tang et al. (2011) have suggested that *Belligobio* is a junior synonym of *Hemibarbus*. Therefore, our phylogenetic analyses included eight mitochondrial genomes from GenBank as follows: *B. nummifer* (KJ413052; Wang et al. 2016), *Hemibarbus medius* (KF640234; Chen et al. 2015), *H. umbrifer* (AP011415; Tang et al. 2011), *H. maculatus* (JF906109; Li et al. 2012), *H. labeo* (DQ347953; Kim et al. 2009), *H. longirostris* (DQ347952; Kim et al. 2009), *Squalidus chankaensis* (MT767746; Chai and Fu, 2020) and *S. multimaculatus* (KX495606; Yi et al. 2017). Our phylogeny was inferred using maximum likelihood (ML) and Bayesian (BI) methods based on 13 protein-coding genes or complete mitochondrial genomes, which the analyses of ML and BI were performed on the software IQ-TREE v2.1.3 (Minh et al. 2020) and BEAST v2.6.7 (Bouckaert and Drummond 2017), respectively. The ML phylogeny was reconstructed with 1000 ultrafast bootstrap replicates (Hoang et al. 2018) and the best models of nucleotide substitution searched by ModelFinder in IQ-TREE. The BI phylogeny was reconstructed with 50,000,000 generations sampled per 1000 generations, and the models of nucleotide substitution were automatically acquired through bModelTest Analyzer in BEAST.

## 4. Results

A reference Image of *B. pengxianensis* is provided in Figure 1. *B. pengxianensis* is mainly distinguished from other species in the Gobionidae by the combination of three morphological characteristics, as follows: Three row teeth, soft and slim of the last simple dorsal ray, and the scales on the chest hidden under the skin (Yue 1998).

**Figure 2.**
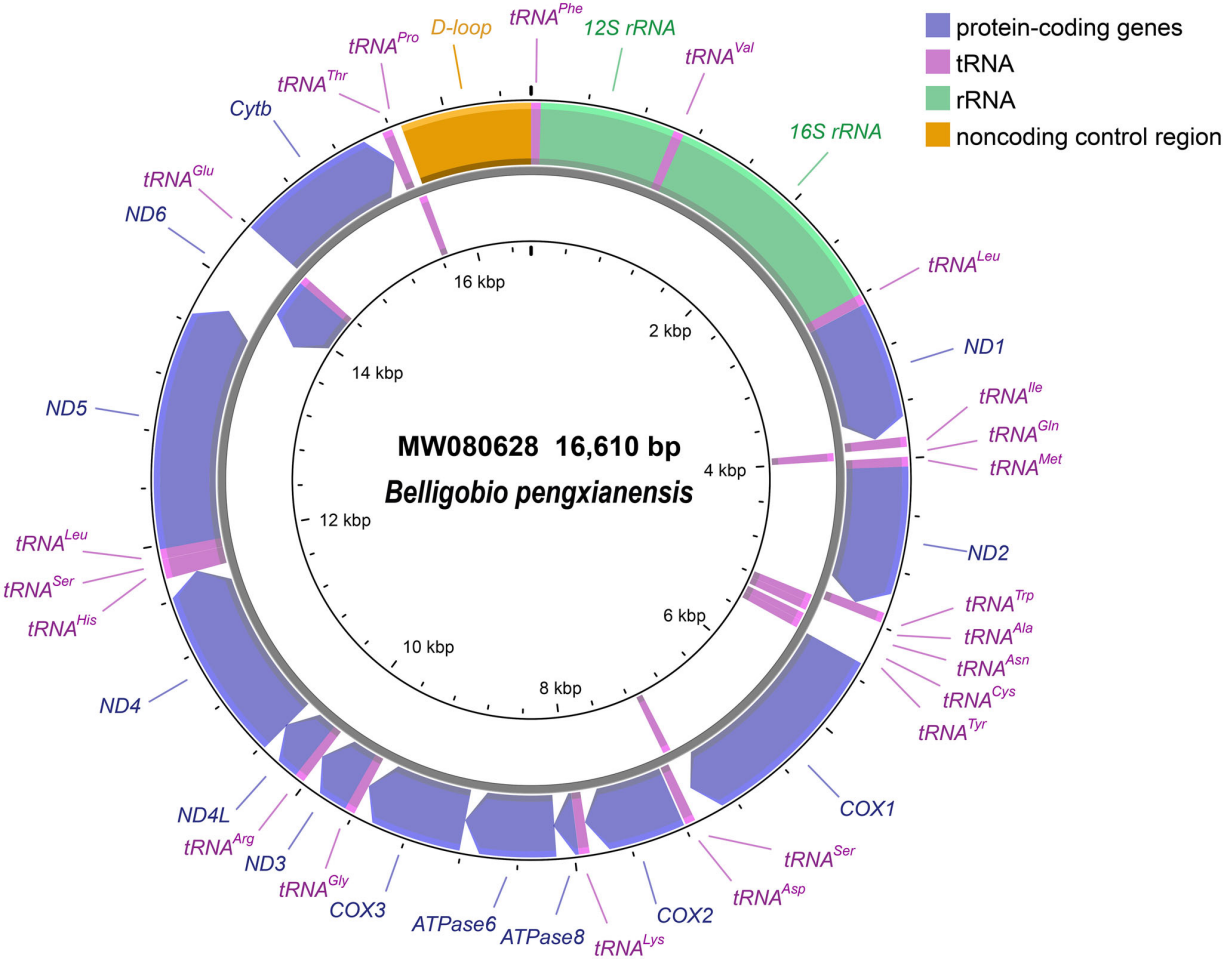
Mitochondrial genome map of *B. pengxianensis.*

The complete mitochondrial genome of *B. pengxianensis* (MW080628) was assembled from PCR products of 13 segments (supplemental Appendixes I and II). This mitogenome is 16,610 bp in length with the AT content of 55.23%. The circular mitogenome consists of 13 protein-coding genes, two rRNA genes, 22 tRNA genes and one non-coding control region (Figure 2). The characteristics of the mitogenome are provided in Table 1. The protein-coding genes start with ATG/GTG and end with TAA/TAG/TA-/T–. Two conserved blocks of the control region (5′-CAAACCCCCCTACCCCCT-3′ and 5′-TGTCAAACCCCGAAACCA-3′) named CSB-II and CSB-III by Lee et al. (1995), are located in 16,370–16,387 bp and 16,412–16,429 bp, respectively.

**Figure 3.**
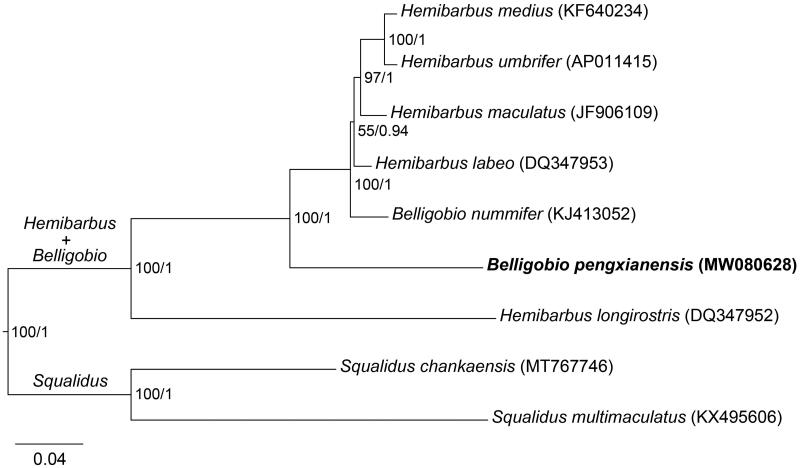
Maximum-likelihood phylogeny between *B. pengxianensis* and its close relatives based on the 13 mitochondrial protein-coding genes. The bootstrap confidences (1000 replicates) and Bayesian posterior probabilities are shown near nodes.

The reconstructed phylogenetic trees display consistent topologies under the analyses of ML and BI using 13 protein-coding genes (Figure 3) or complete mitochondrial genomes (supplemental Figure S2 in Appendix III). The results show that *B. pengxianensis* and *B. nummifer* don’t form the sister relationships, and all of them are nested within the genus *Hemibarbus* with high support values.

## 5. Discussion and conclusion

In this study, we report the mitochondrial genome of *B. pengxianensis* for the first time. This mitogenome displays the same patterns in the gene compositions, gene arrangements, A + T bias of base compositions, the use of start or stop codon, and conserved sequence blocks of the control region as *B. nummifer* (Wang et al. 2016) and other gobionid species (see, e.g. Kim et al. 2009; Li et al. 2012; Chai and Fu 2020; Tian et al. 2022). The phylogenetic nesting of *B. pengxianensis* and *B. nummifer* in the genus *Hemibarbus* was revealed in our phylogeny, bringing into question the validity of the genus *Belligobio*. In summary, the new mitogenome of *B. pengxianensis* could contribute to reconstructing inter-specific relationships among species of the genera *Hemibarbus* and *Belligobio*, and is also very useful as reference molecular information to support species identification, biodiversity monitoring and conservation in the future.

## Supplementary Material

Supplemental Material

Supplemental Material

Supplemental Material

## Data Availability

The genome sequence data that support the findings of this study are openly available in GenBank of NCBI at https://www.ncbi.nlm.nih.gov/ under the accession no.MW080628. Sanger sequencing results are provided in supplementary materials.
