# Supplementary material for: Complete mitochondrial genome of Belligobio pengxianensis (Cypriniformes: Gobionidae)
Source: Mitochondrial DNA B Resour. 2023 Mar 25;8(3):434–8. doi: 10.1080/23802359.2023.2192310 (PMC10044147; doi:10.1080/23802359.2023.2192310)
Supplement: Supplemental Material [file TMDN_A_2192310_SM6997.docx]

**Supplementary Material—Appendix III**

**Complete mitochondrial genome of *Belligobio pengxianensis* (Cypriniformes: Gobionidae)**

Bo Xuan, Mingyue Li, Xiaomin Ni and Cuizhang Fu*


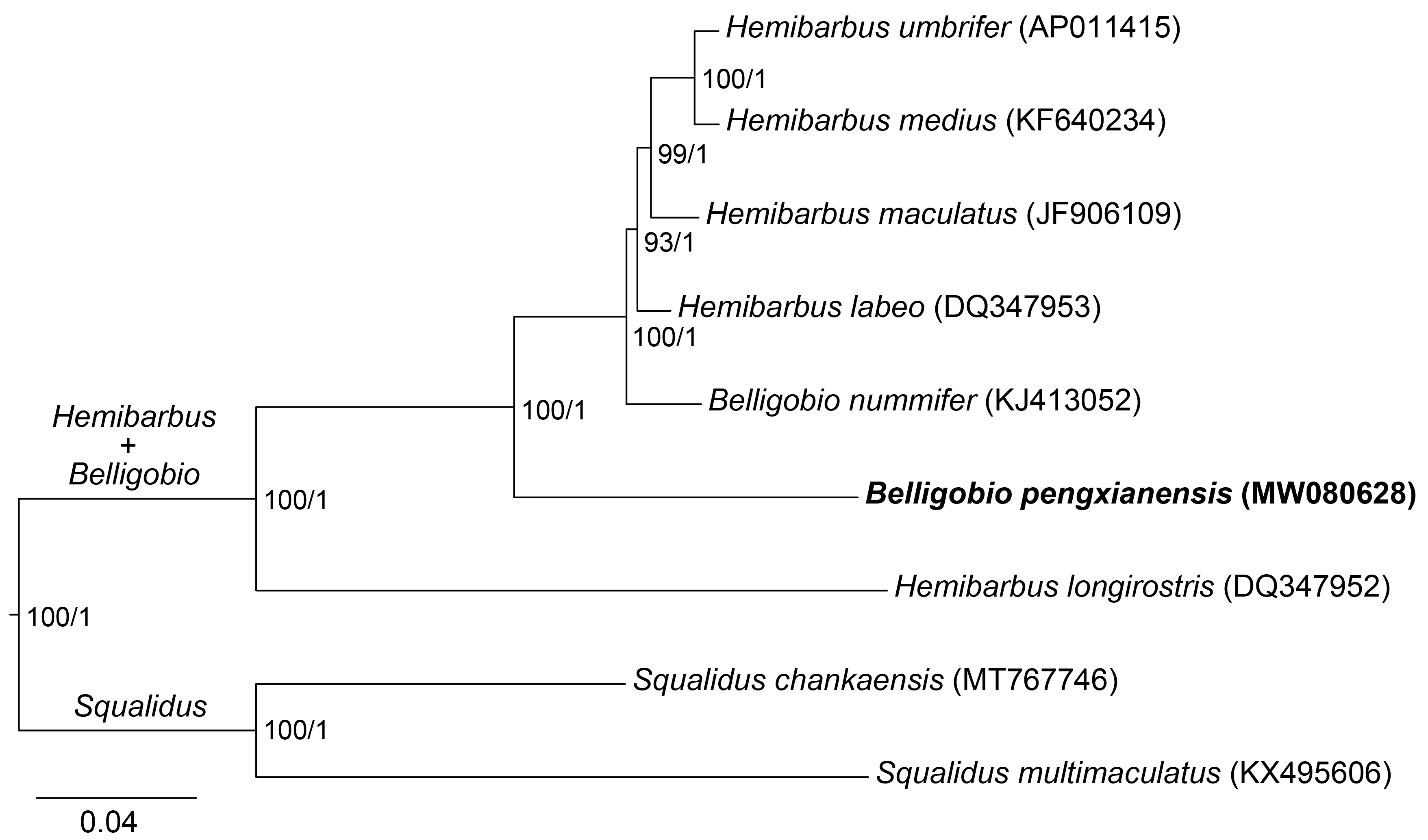


Figure S2 Maximum-likelihood phylogeny between *B. pengxianensis* and its close relatives based on the complete mitochondrial genome dataset. The bootstrap conﬁdences (1000 replicates) and Bayesian posterior probabilities are shown near nodes.
