# Supplementary material for: Complete mitochondrial genome of Belligobio pengxianensis (Cypriniformes: Gobionidae)
Source: Mitochondrial DNA B Resour. 2023 Mar 25;8(3):434–8. doi: 10.1080/23802359.2023.2192310 (PMC10044147; doi:10.1080/23802359.2023.2192310)

**Supplementary Material—Appendix II**

**Appendix II Raw Sanger sequencing results of fragment 1–13 of the *Belligobio pengxianensis* mitogenome**

1. Fragment 1 was sequenced using primer pair 1.

Fragment 1-anterior sequence (forward sequencing)


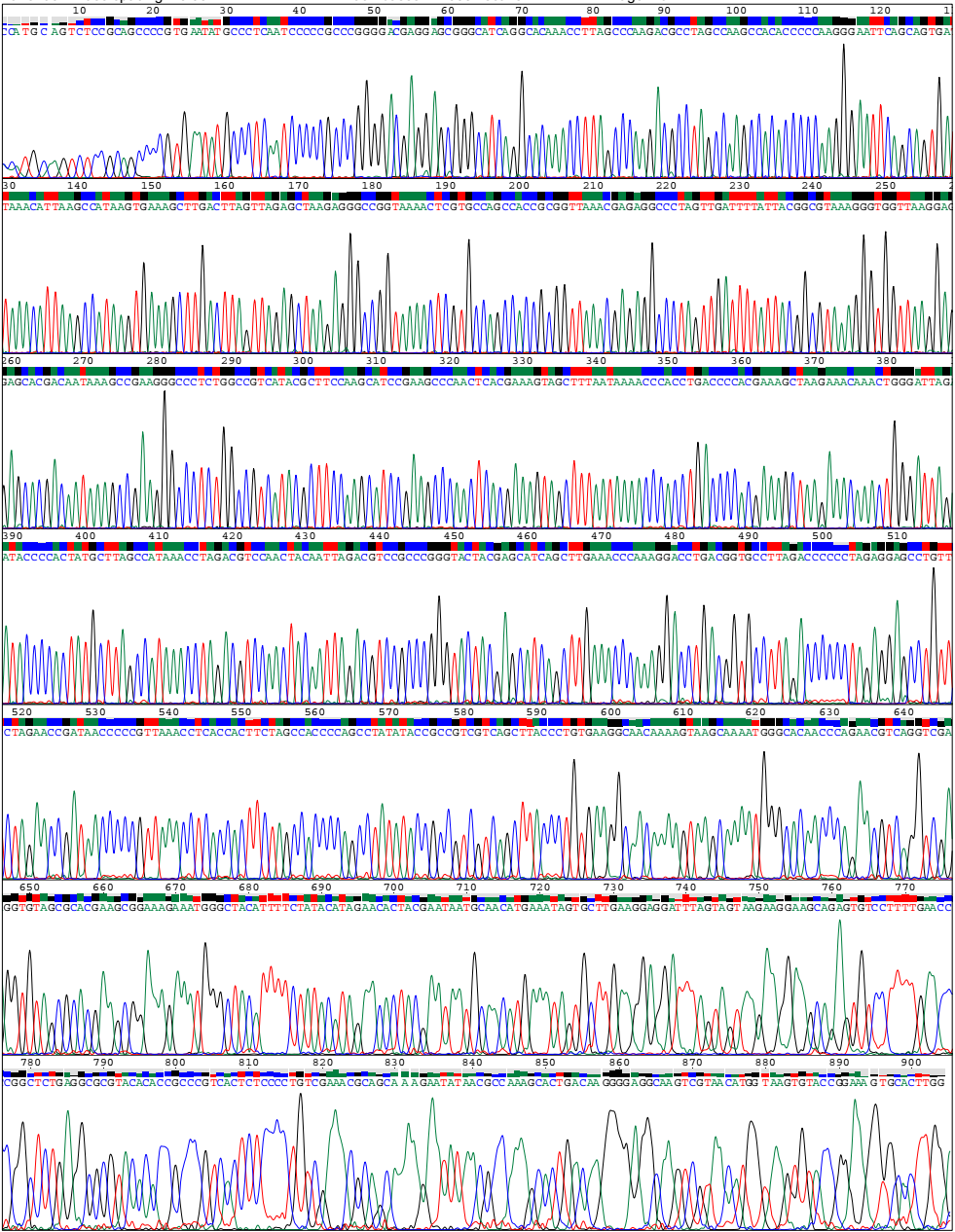


Fragment 1-posterior sequence (reverse sequencing)


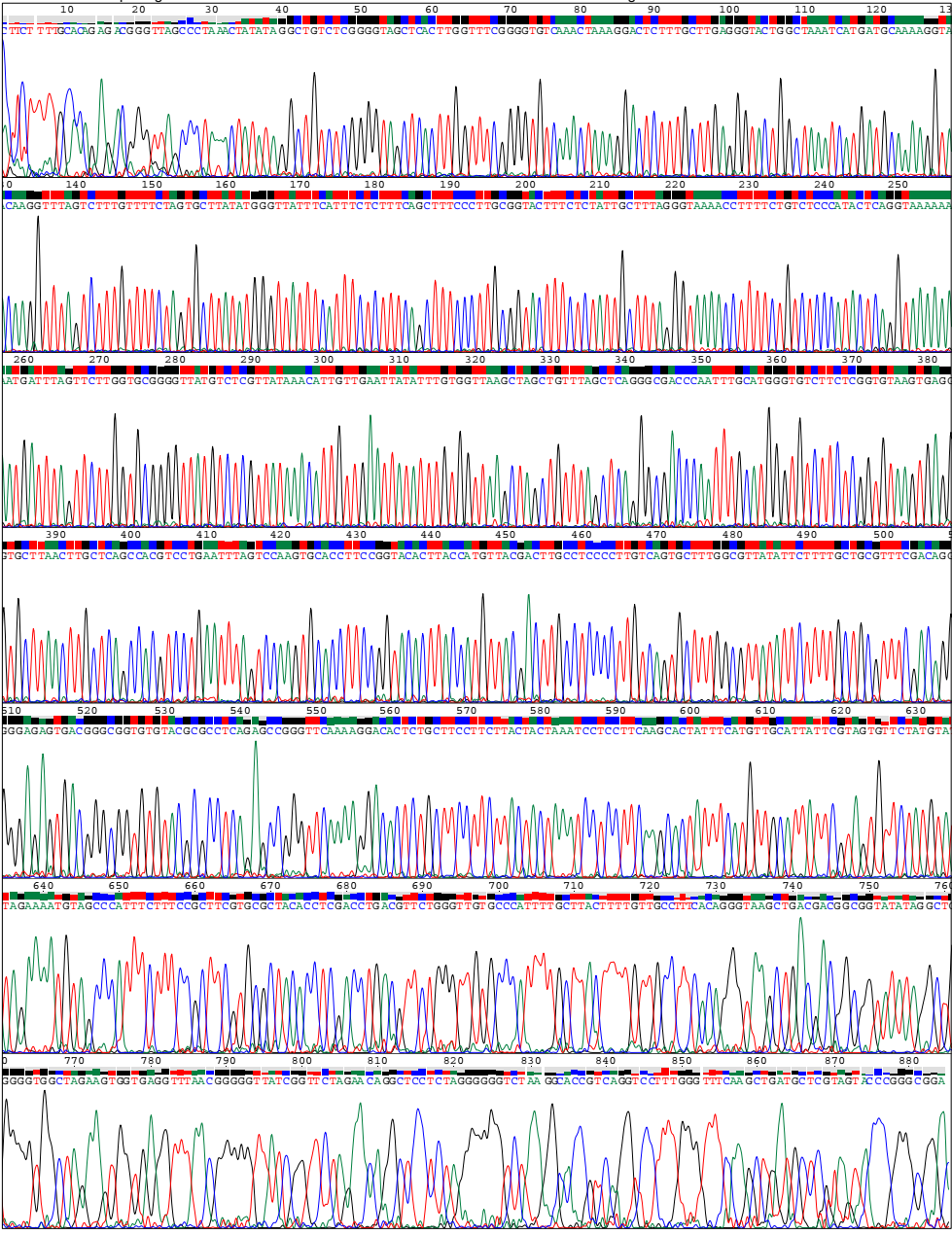


1. Fragment 2 was sequenced using primer pair 2.

Fragment 2-anterior sequence (forward sequencing)


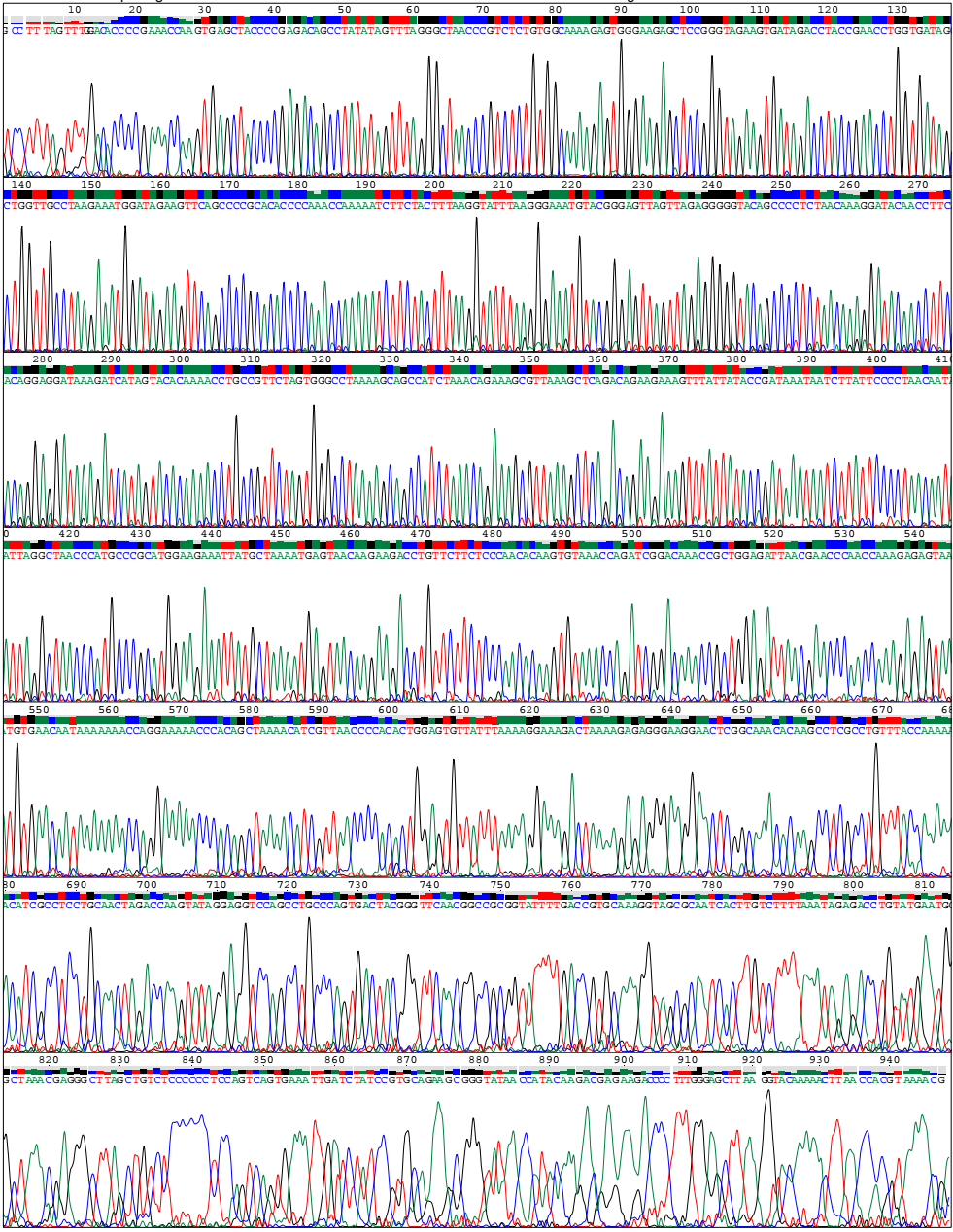


Fragment 2-posterior sequence (reverse sequencing)


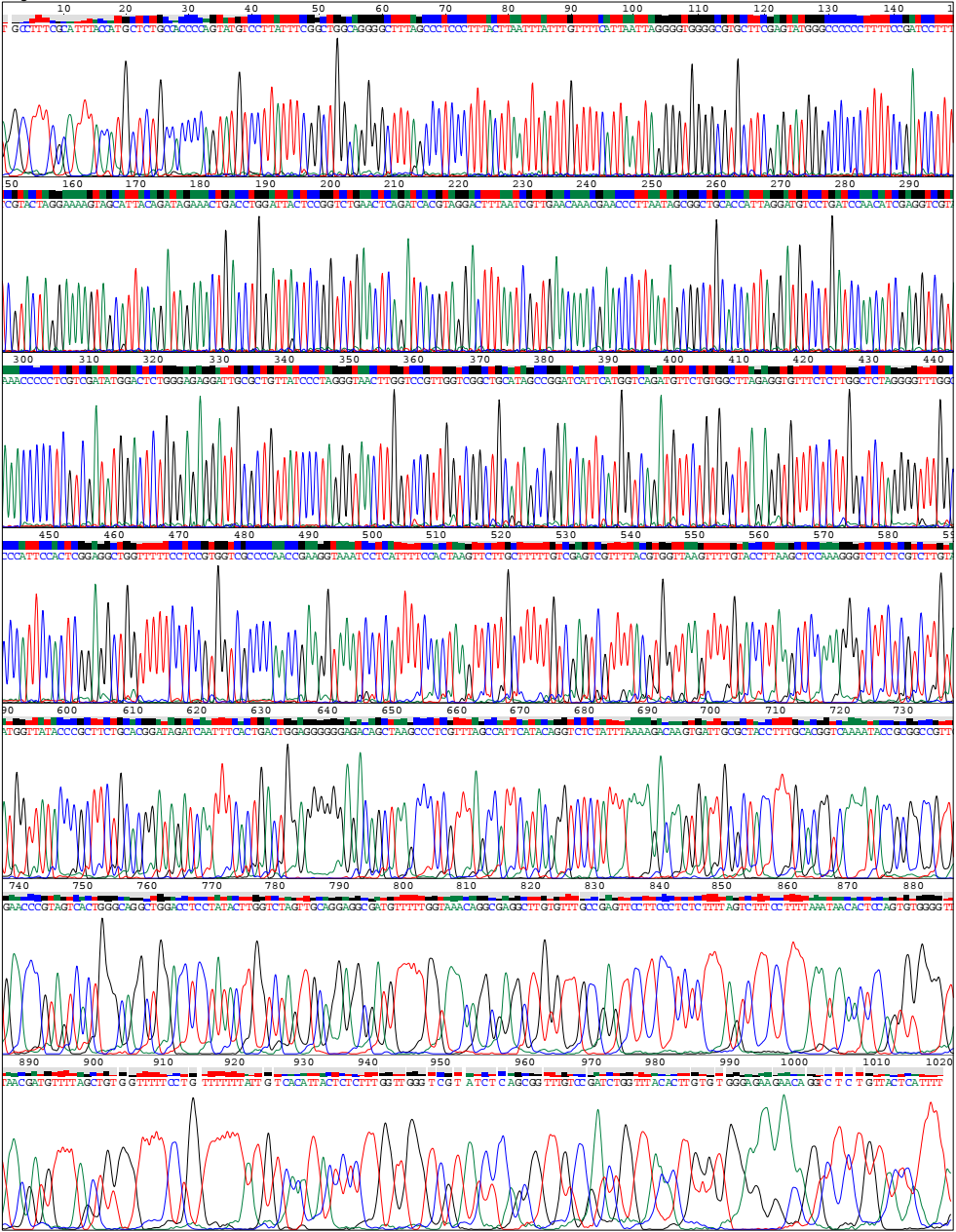


1. Fragment 3 was sequenced using primer pair 3.

Fragment 3-anterior sequence (forward sequencing)


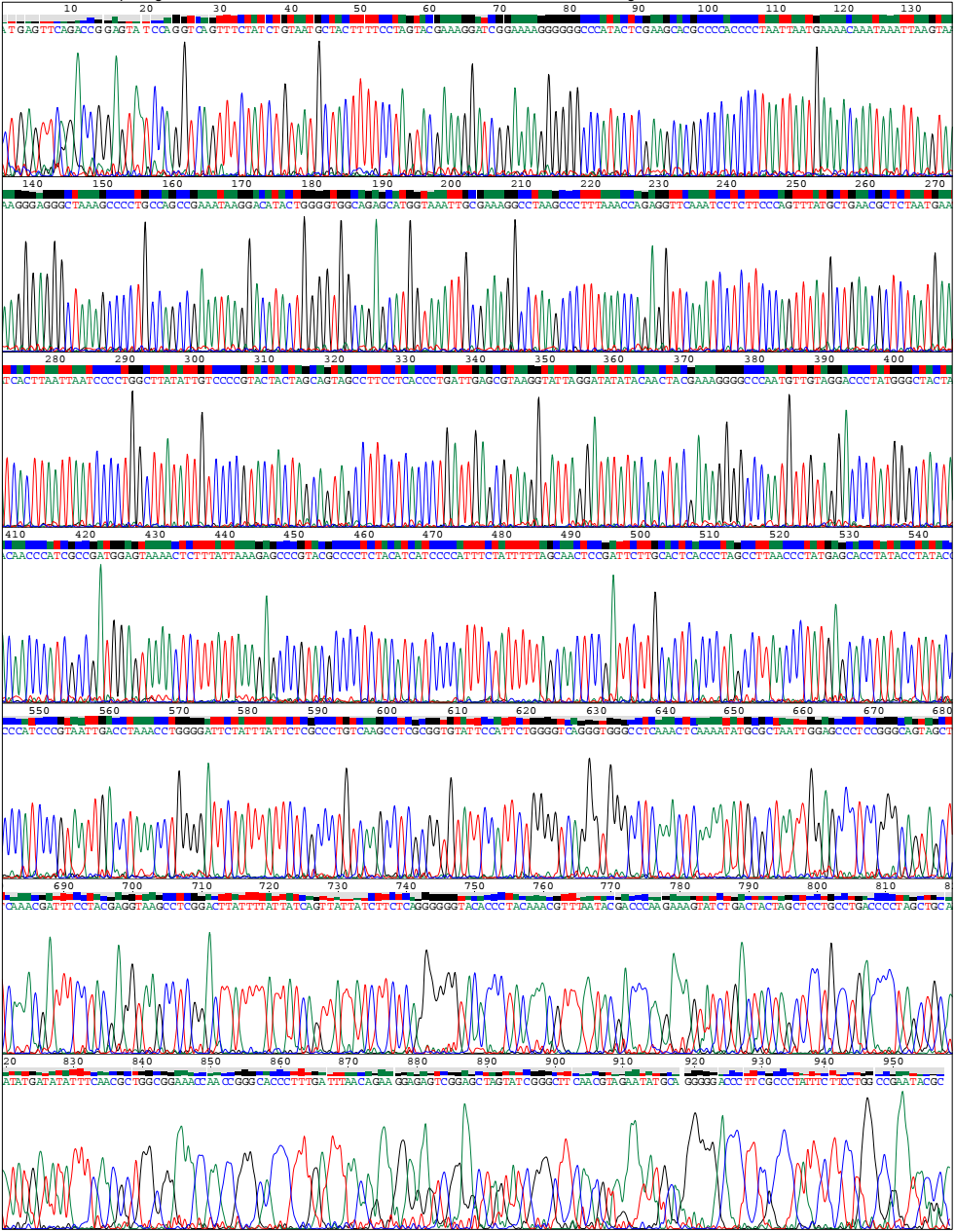


Fragment 3-posterior sequence (reverse sequencing)


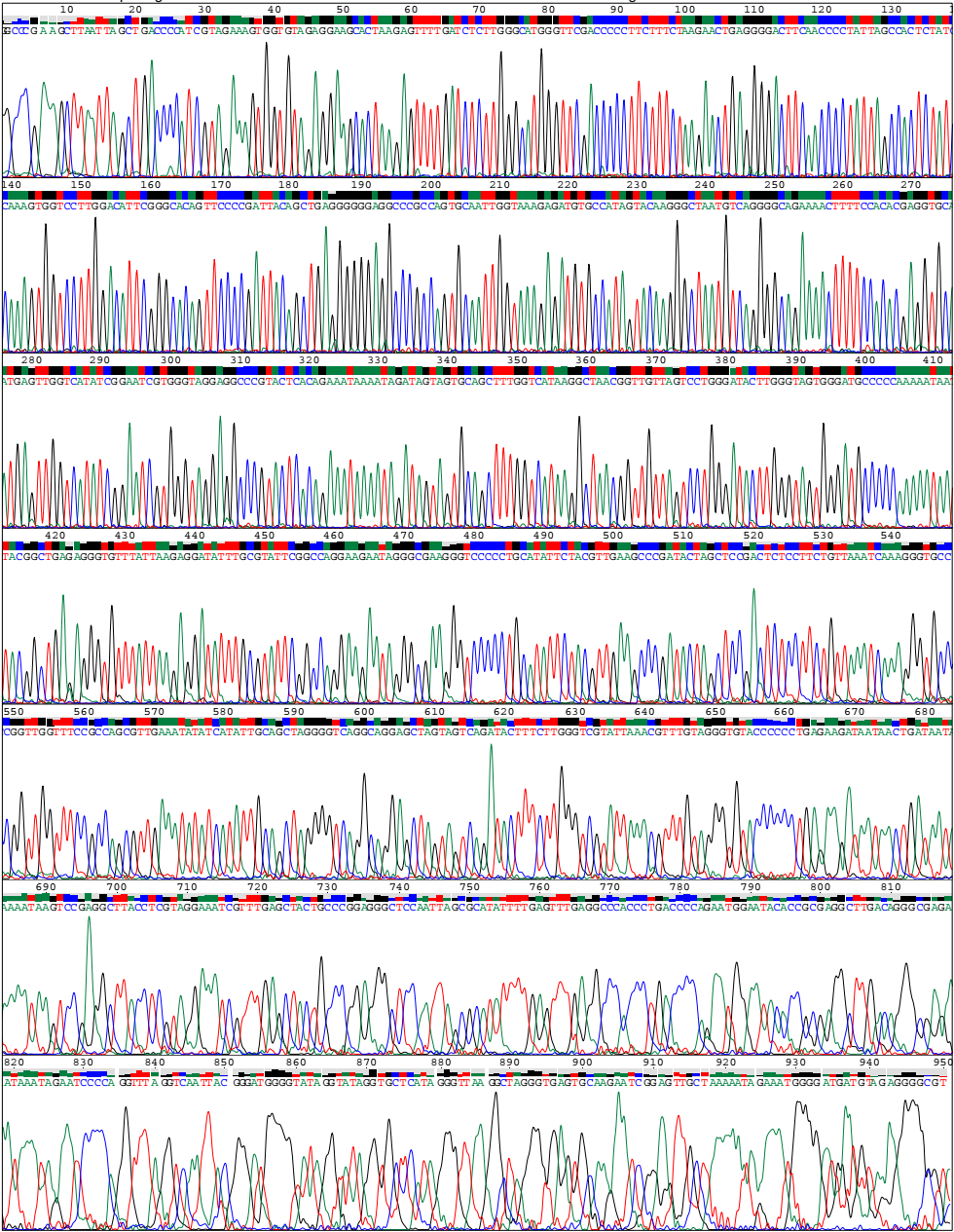


1. Fragment 4 was sequenced using primer pair 4.

Fragment 4-anterior sequence (forward sequencing)


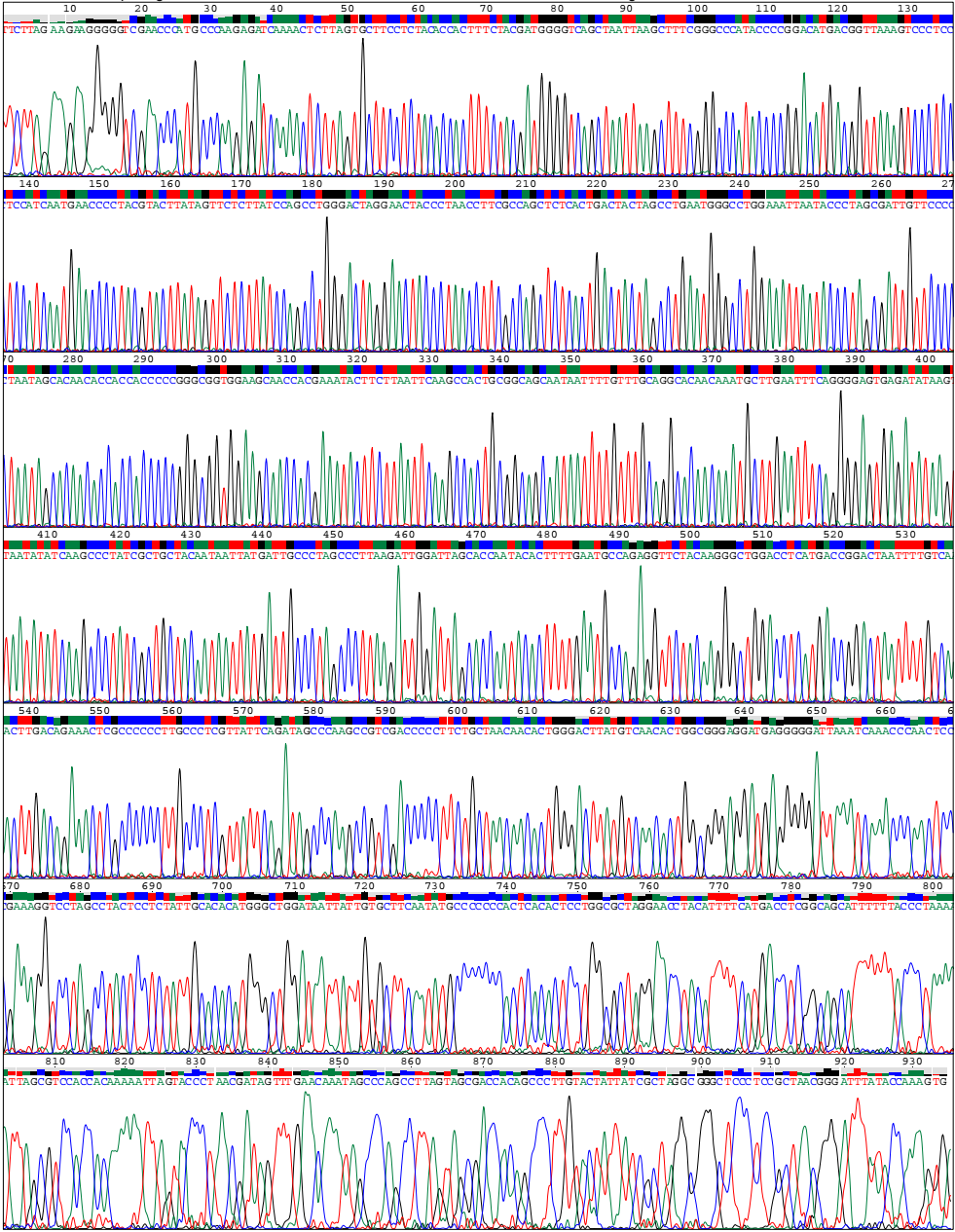


Fragment 4-posterior sequence (reverse sequencing)


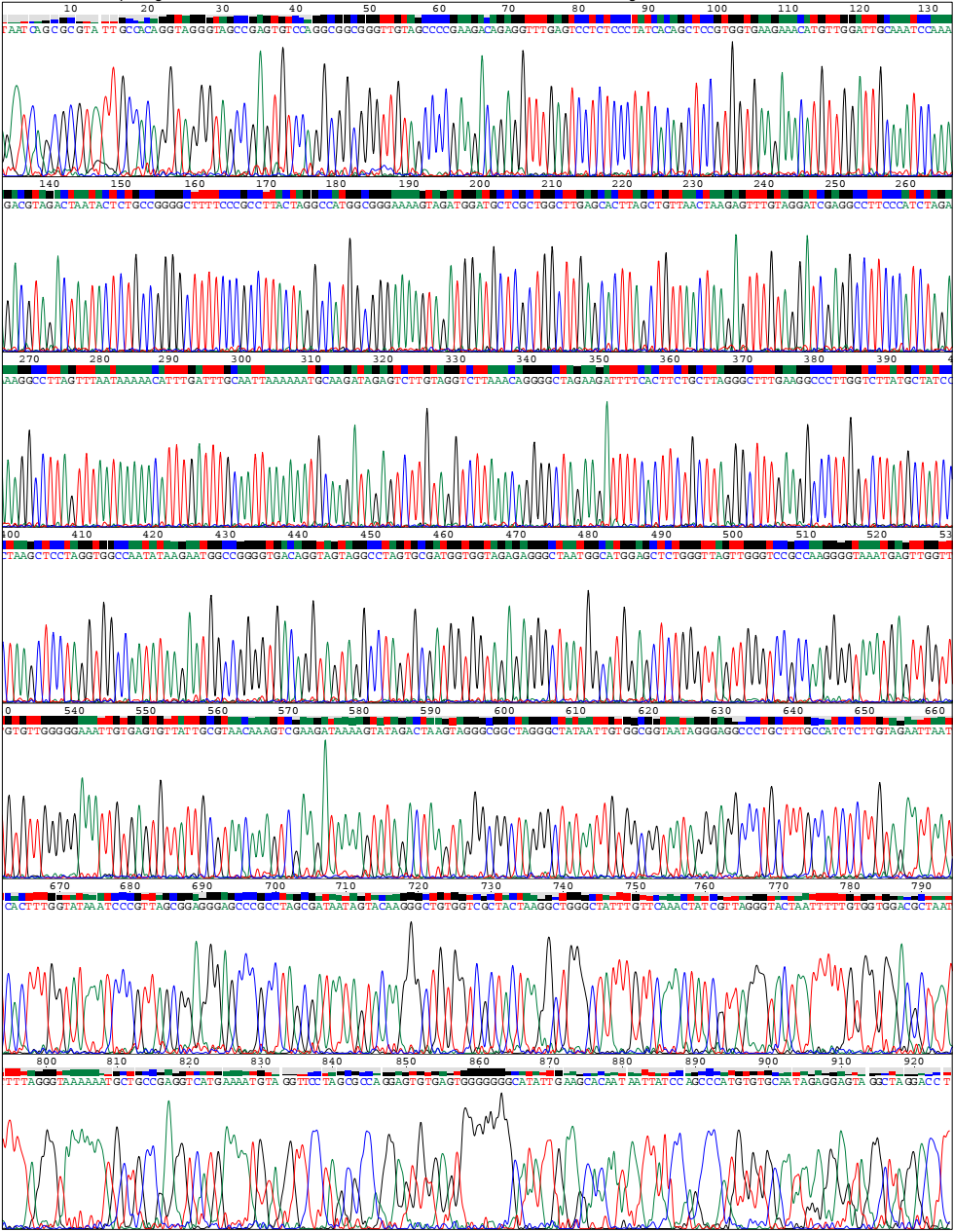


1. Fragment 5 was sequenced using primer pair 5.

Fragment 5-anterior sequence (forward sequencing)


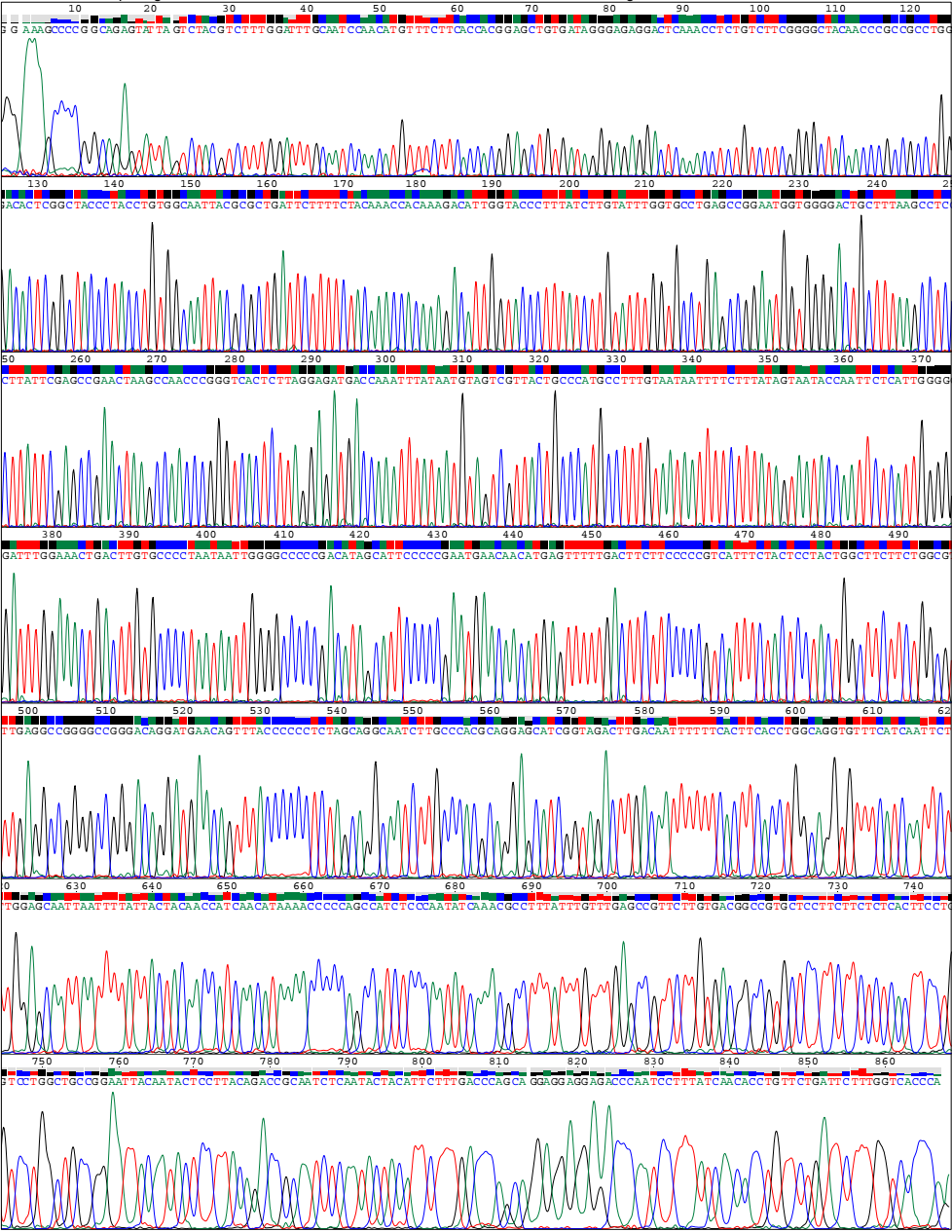


Fragment 5-posterior sequence (reverse sequencing)


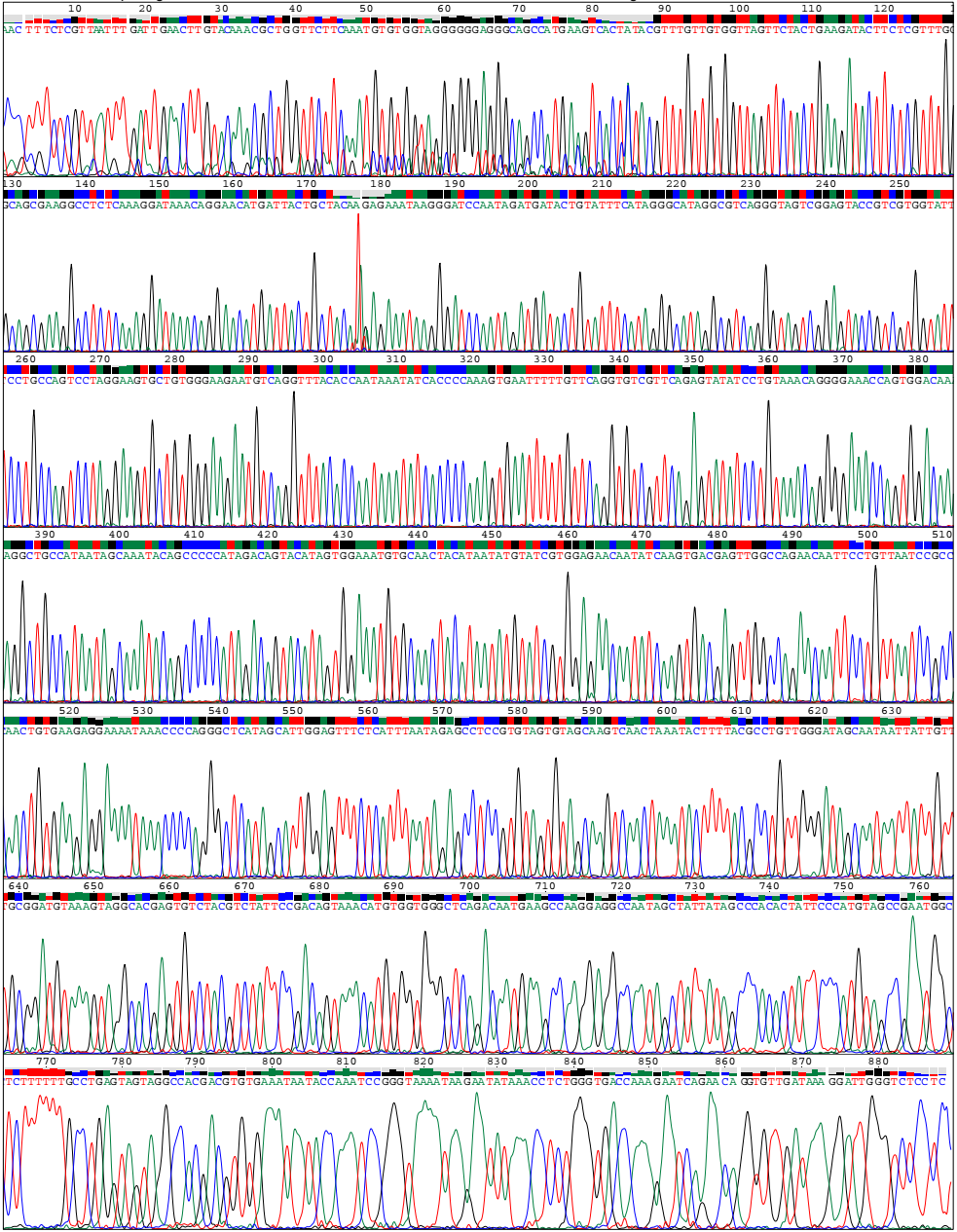


1. Fragment 6-anterior sequence was sequenced using the intermediate primer MeLATP8R (5'-ACTAGAGGTGGTCGGKAGTCA-3') from Tian et al. (2022), and posterior sequence was sequenced using the reverse primer of primer pair 6. [Tian W, Ni XM, Fu CZ. 2022. The complete mitochondrial genome of *Mesogobio lachneri* (Cypriniformes: Gobionidae) from Northeast Asia. Mitochondrial DNA B, 7(10): 1810-1813.]

Fragment 6-anterior sequence (reverse sequencing)


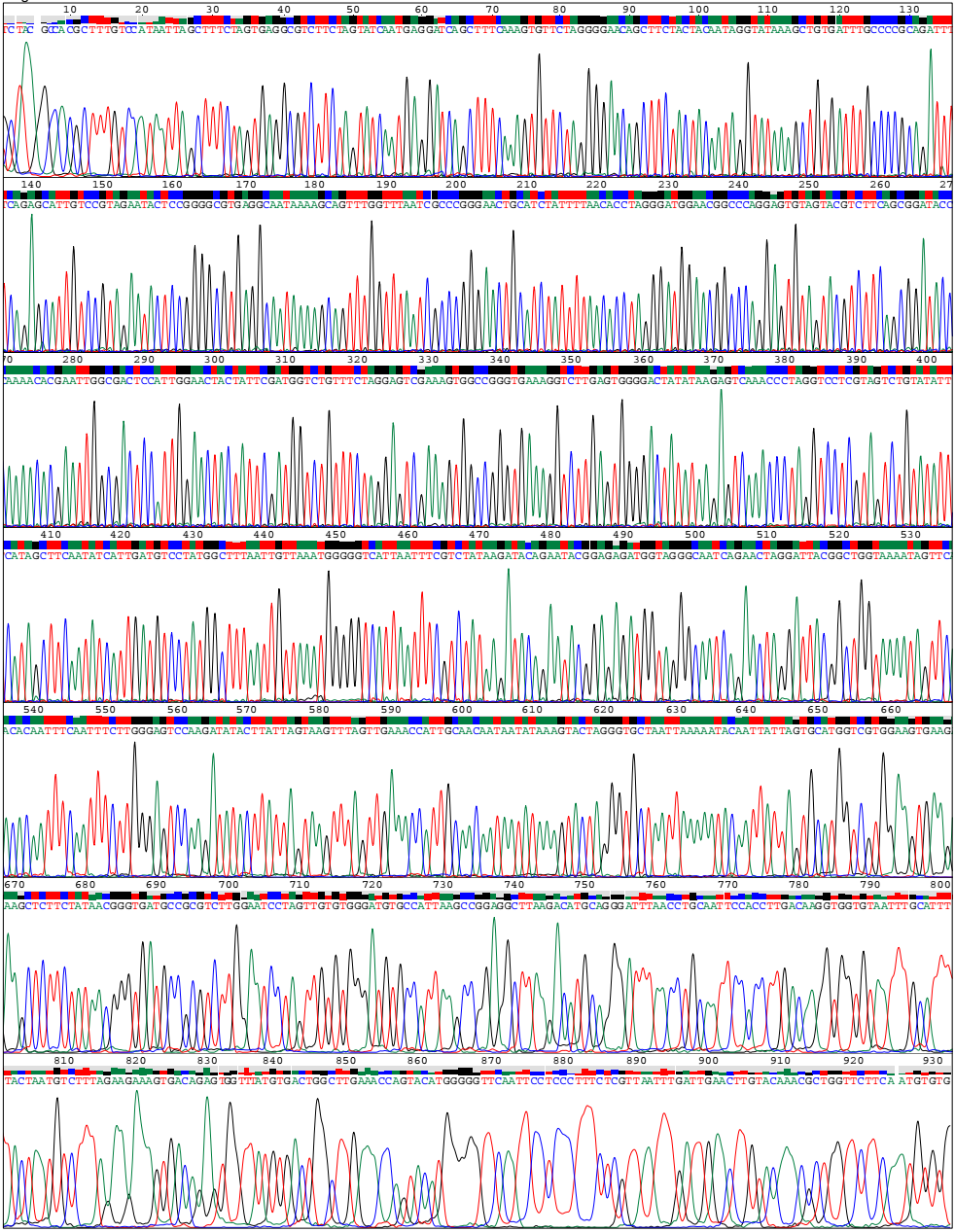


Fragment 6-posterior sequence (reverse sequencing)


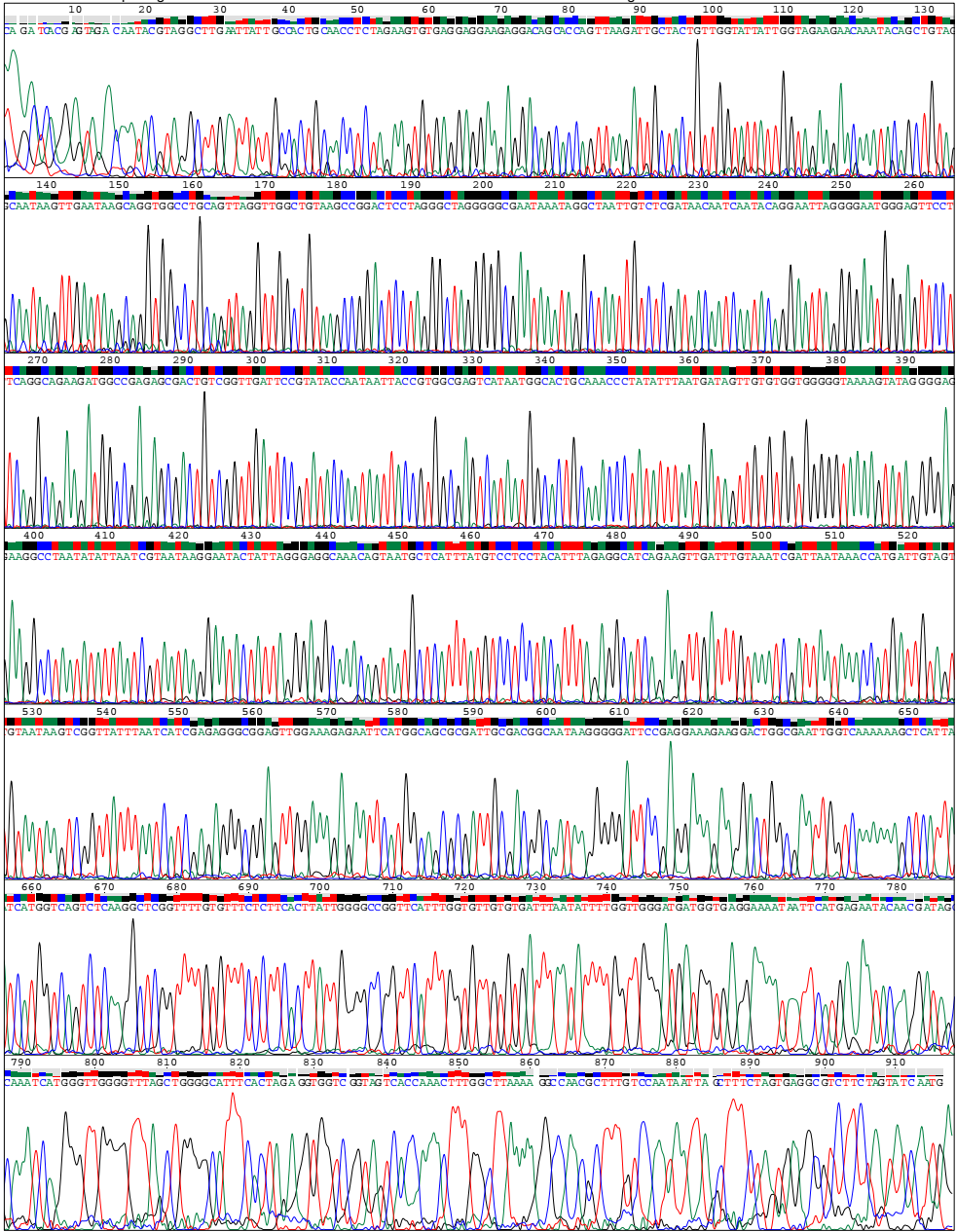


1. Fragment 7 was sequenced using primer pair 7.

Fragment 7-anterior sequence (forward sequencing)


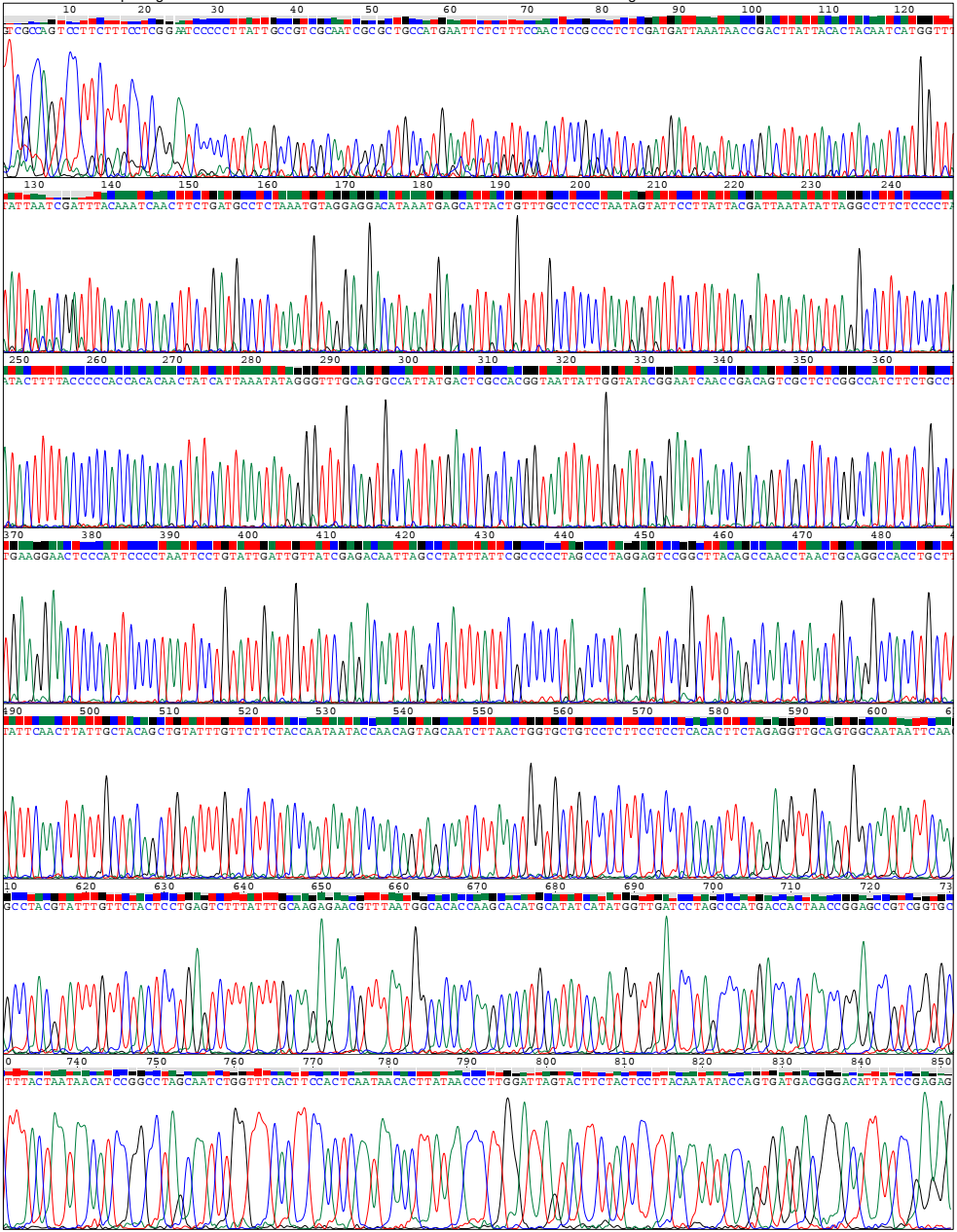


Fragment 7-posterior sequence (reverse sequencing)


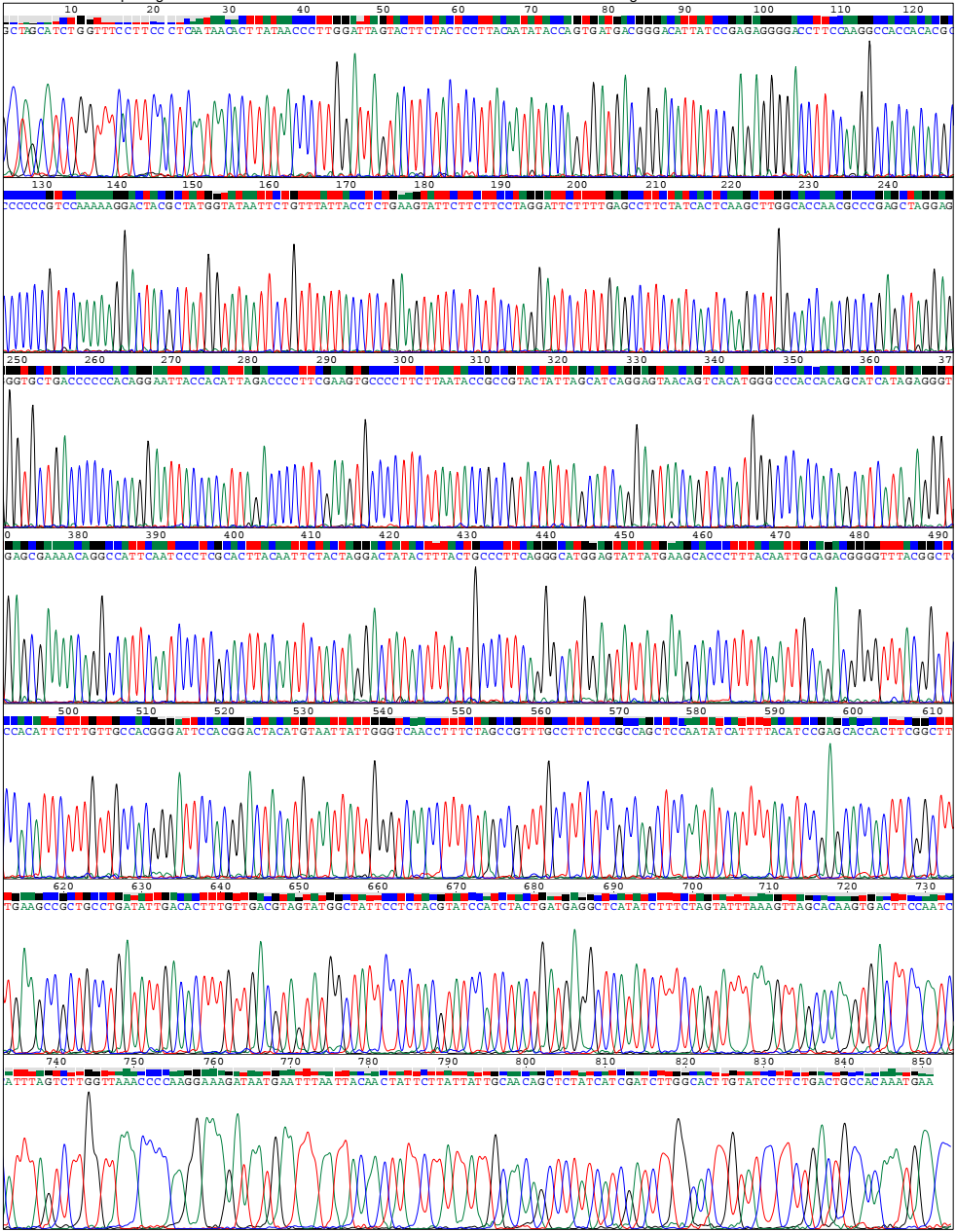


1. Fragment 8 was sequenced using primer pair 8.

Fragment 8-anterior sequence (forward sequencing)


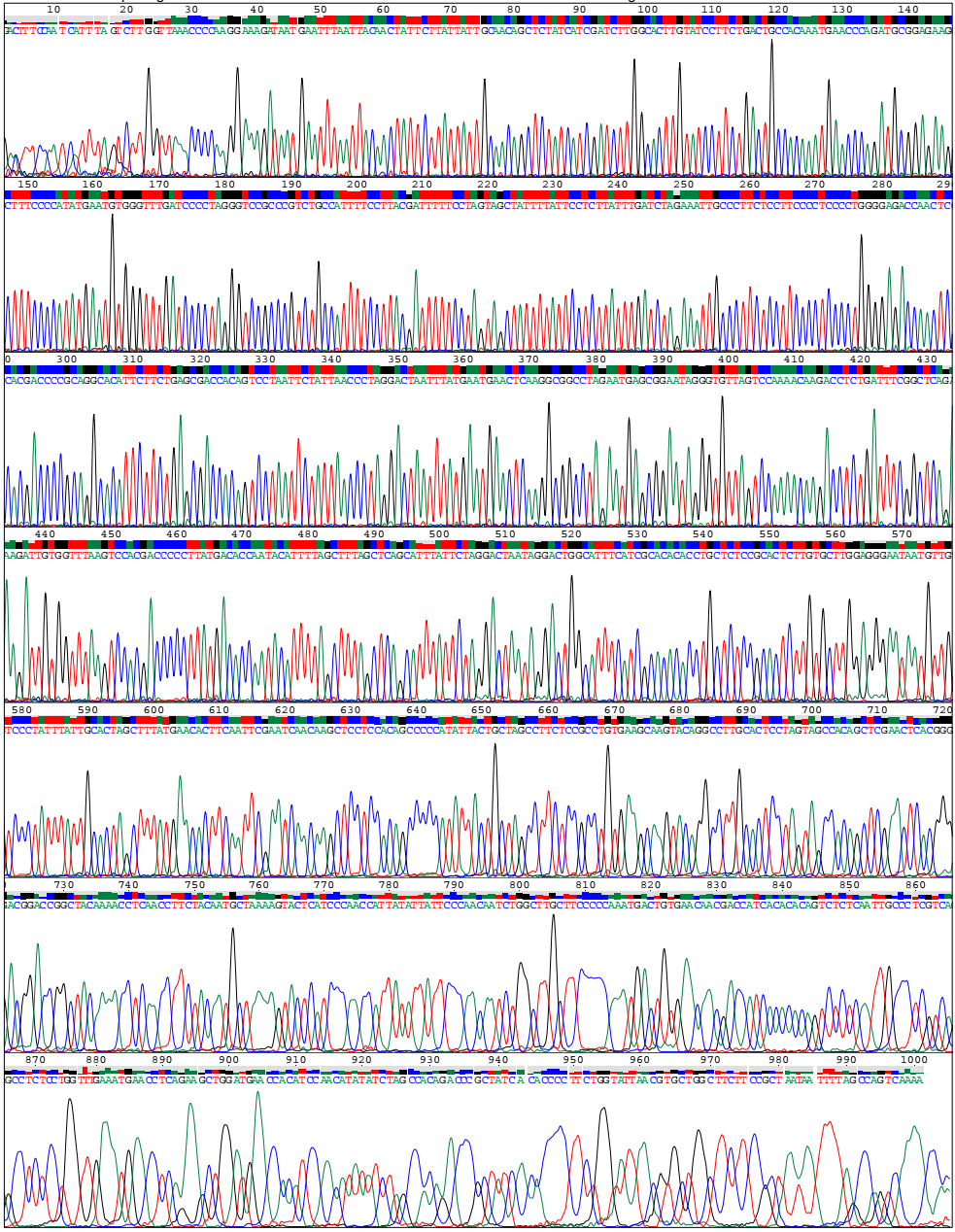


Fragment 8-posterior sequence (reverse sequencing)


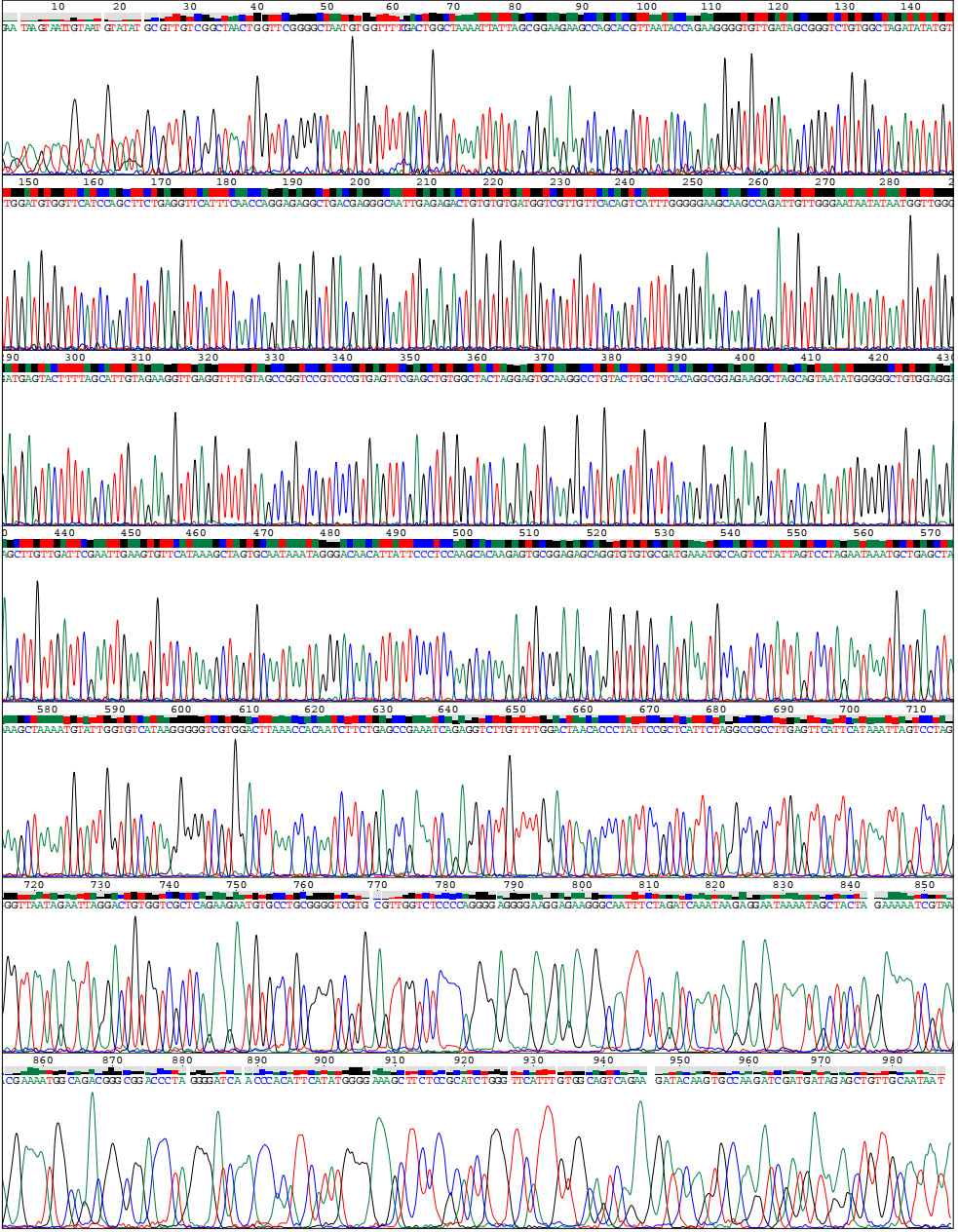


1. Fragment 9-anterior sequence was sequenced using the intermediate primer BPND4R (5'-CCTGCTACRAGKCCTATGTG-3'), and posterior sequence was sequenced using the reverse primer of primer pair 9.

Fragment 9-anterior sequence (reverse sequencing)


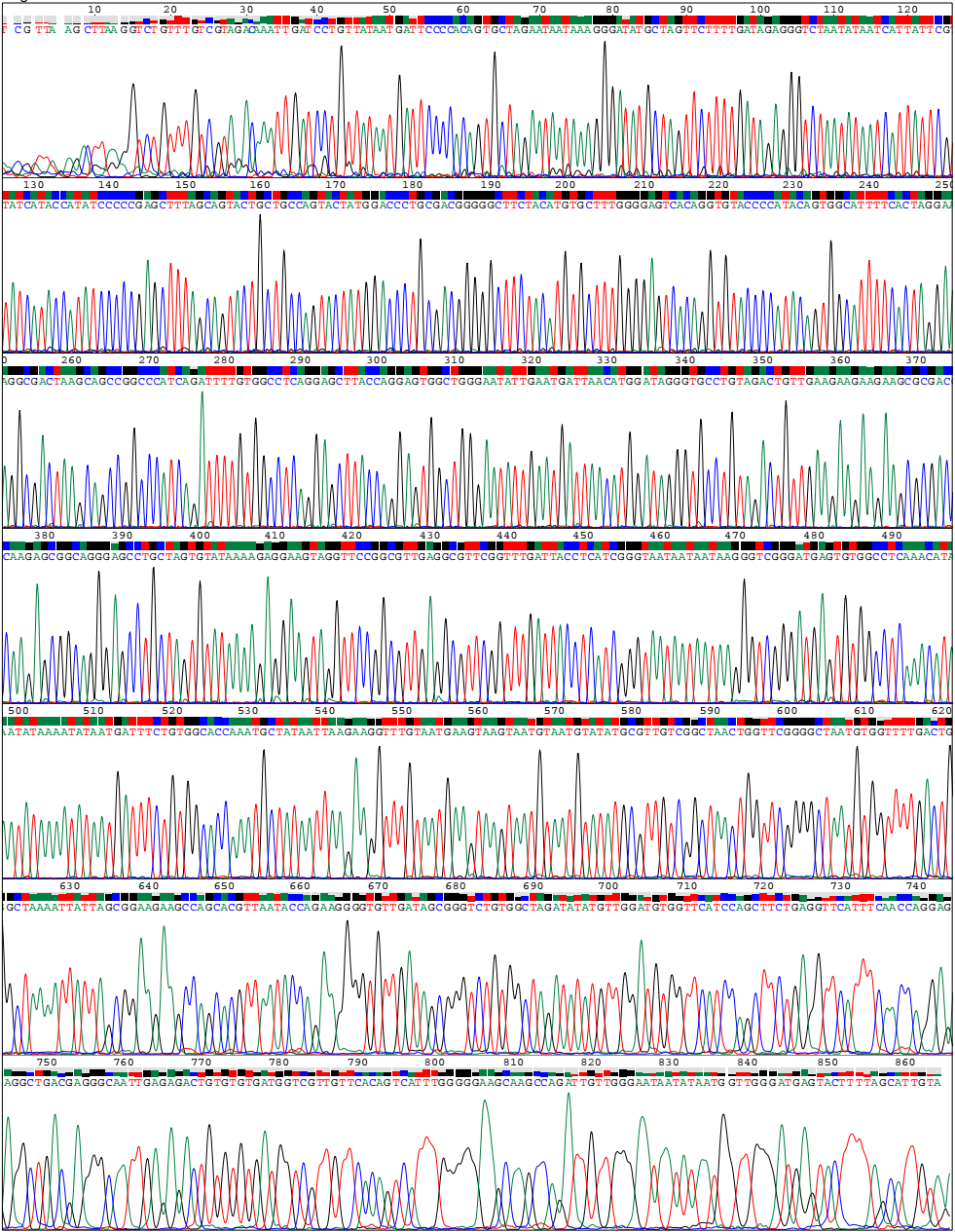


Fragment 9-posterior sequence (reverse sequencing)


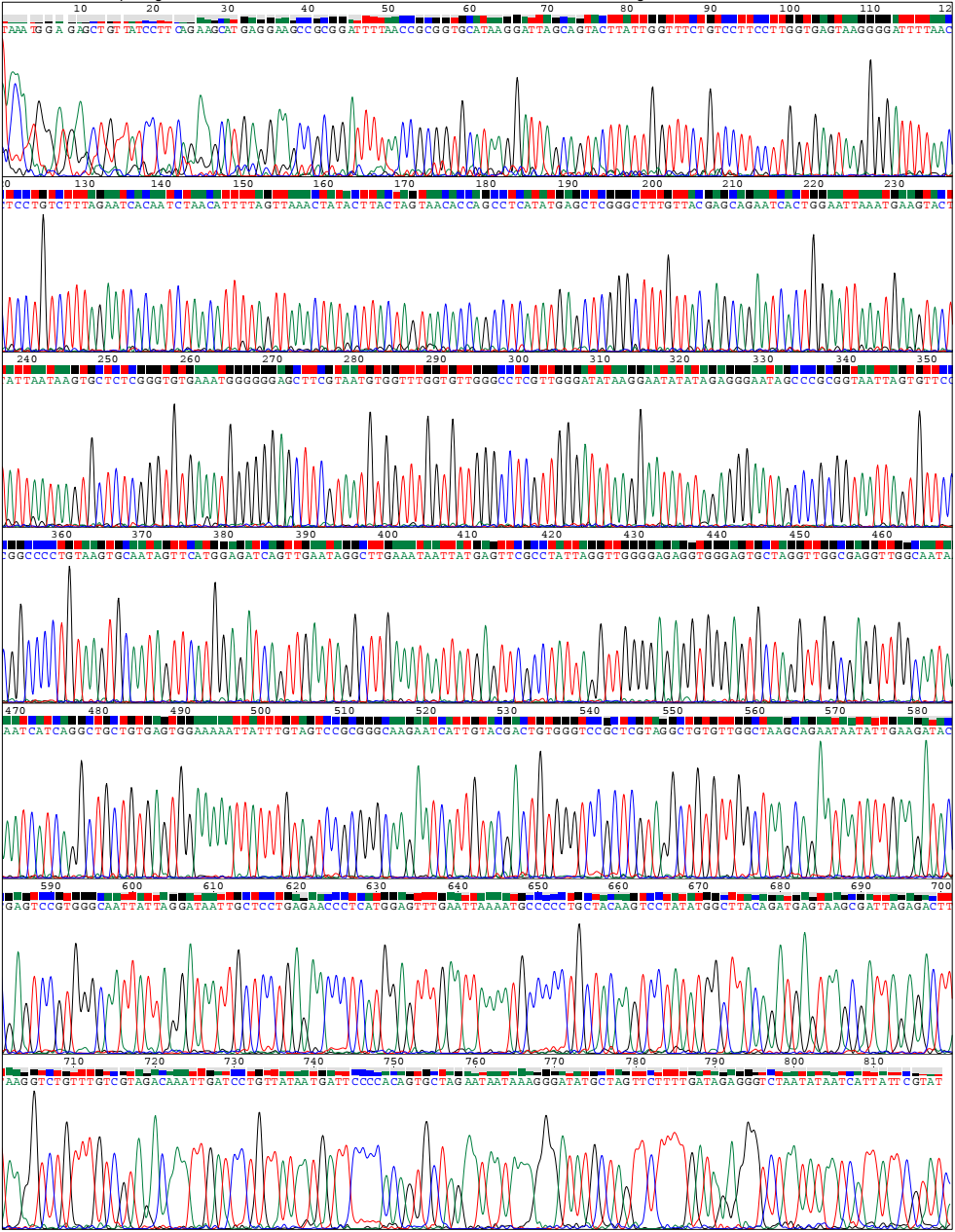


1. Fragment 10 was sequenced using primer pair 10.

Fragment 10-anterior sequence (forward sequencing)


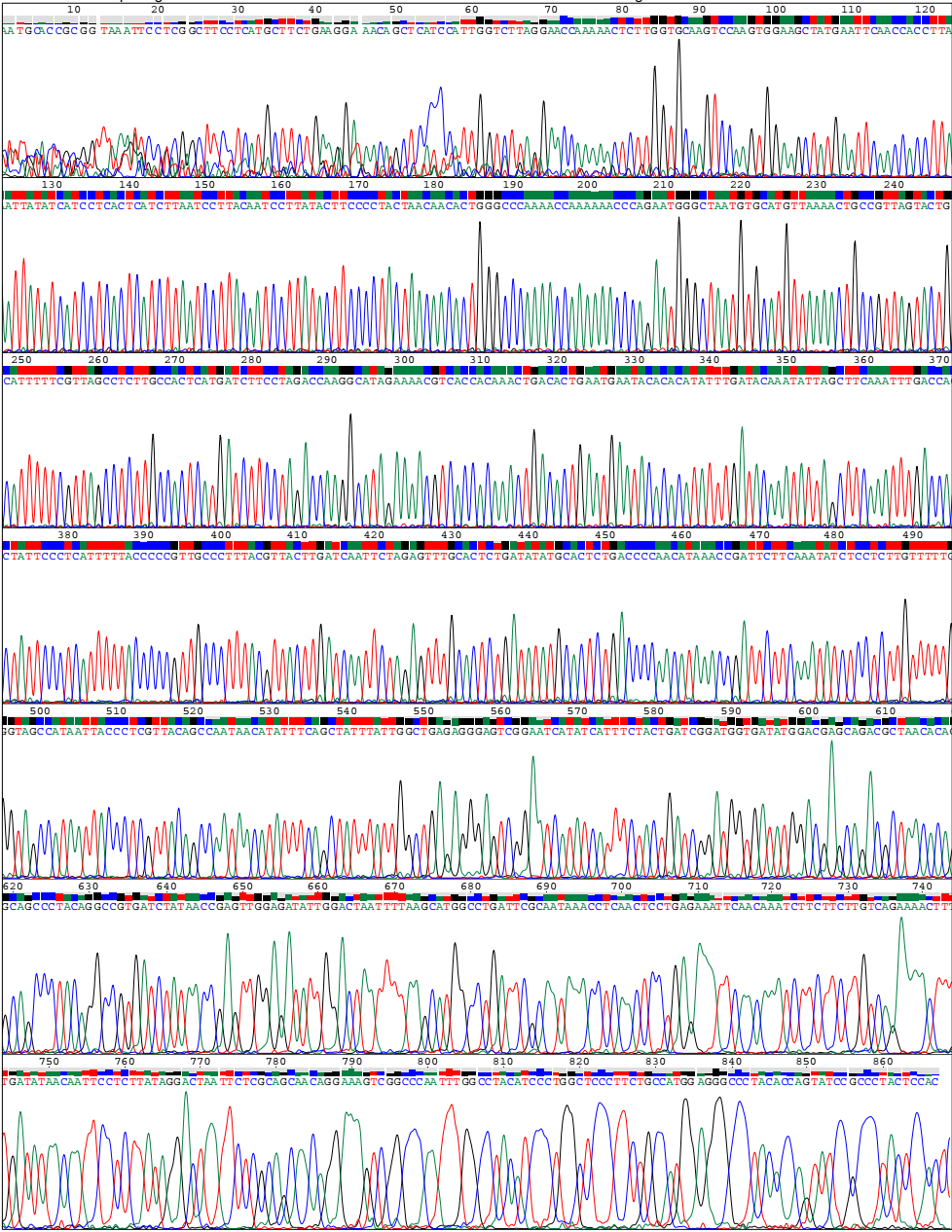


Fragment 10-posterior sequence (reverse sequencing)


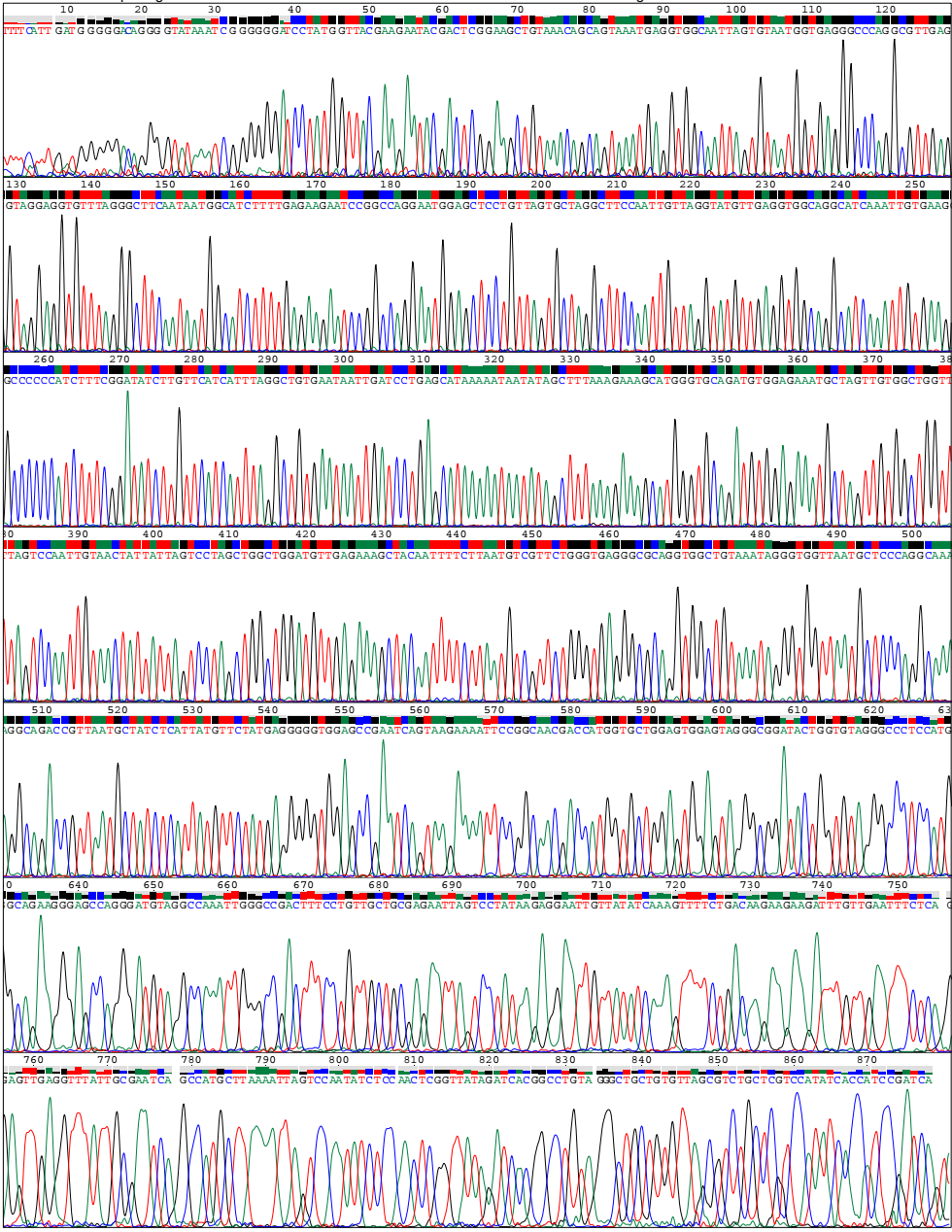


1. Fragment 11 was sequenced using primer pair 11.

Fragment 11-anterior sequence (forward sequencing)


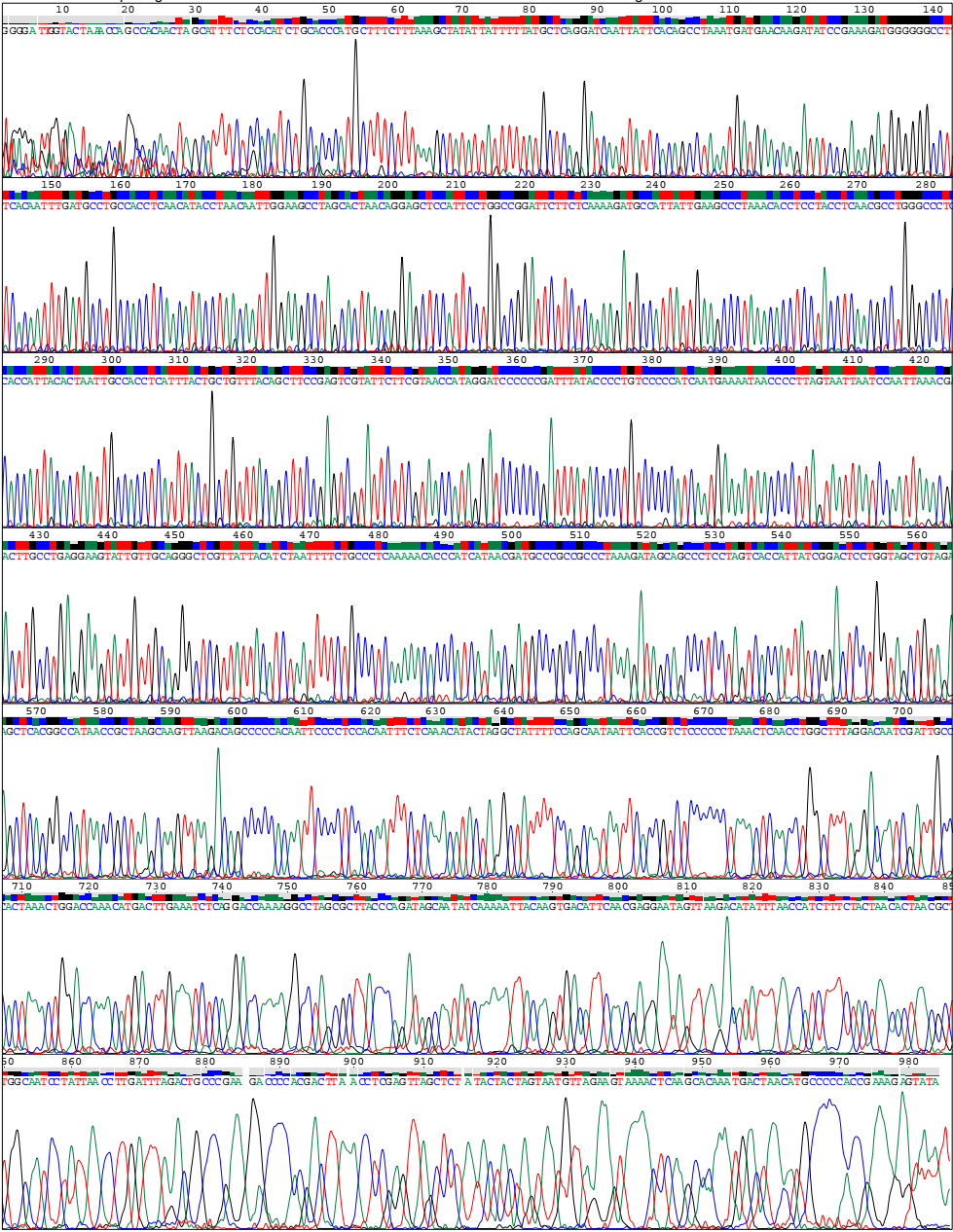


Fragment 11-posterior sequence (reverse sequencing)


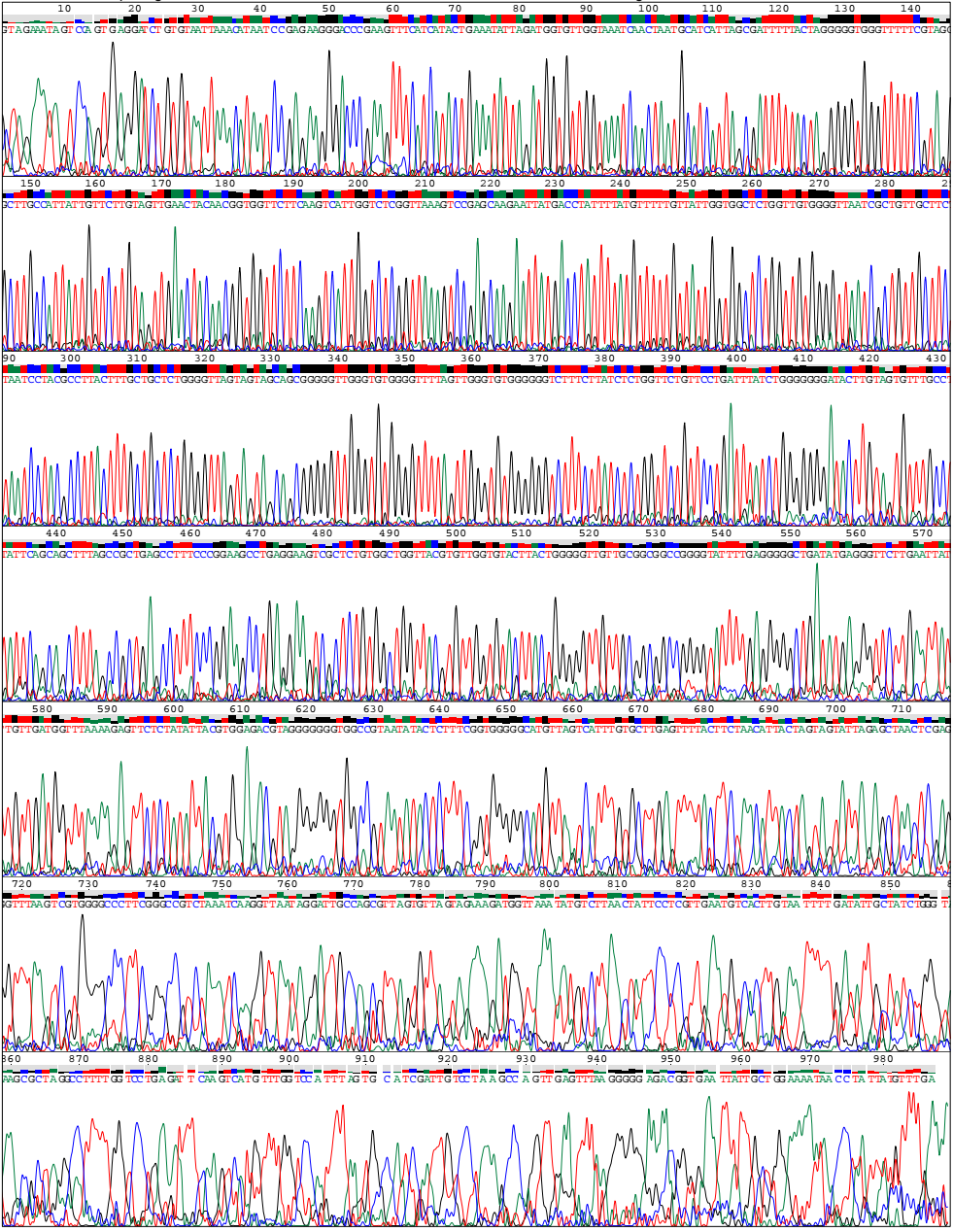


1. Fragment 12 was sequenced using primer pair 12.

Fragment 12-anterior sequence (forward sequencing)


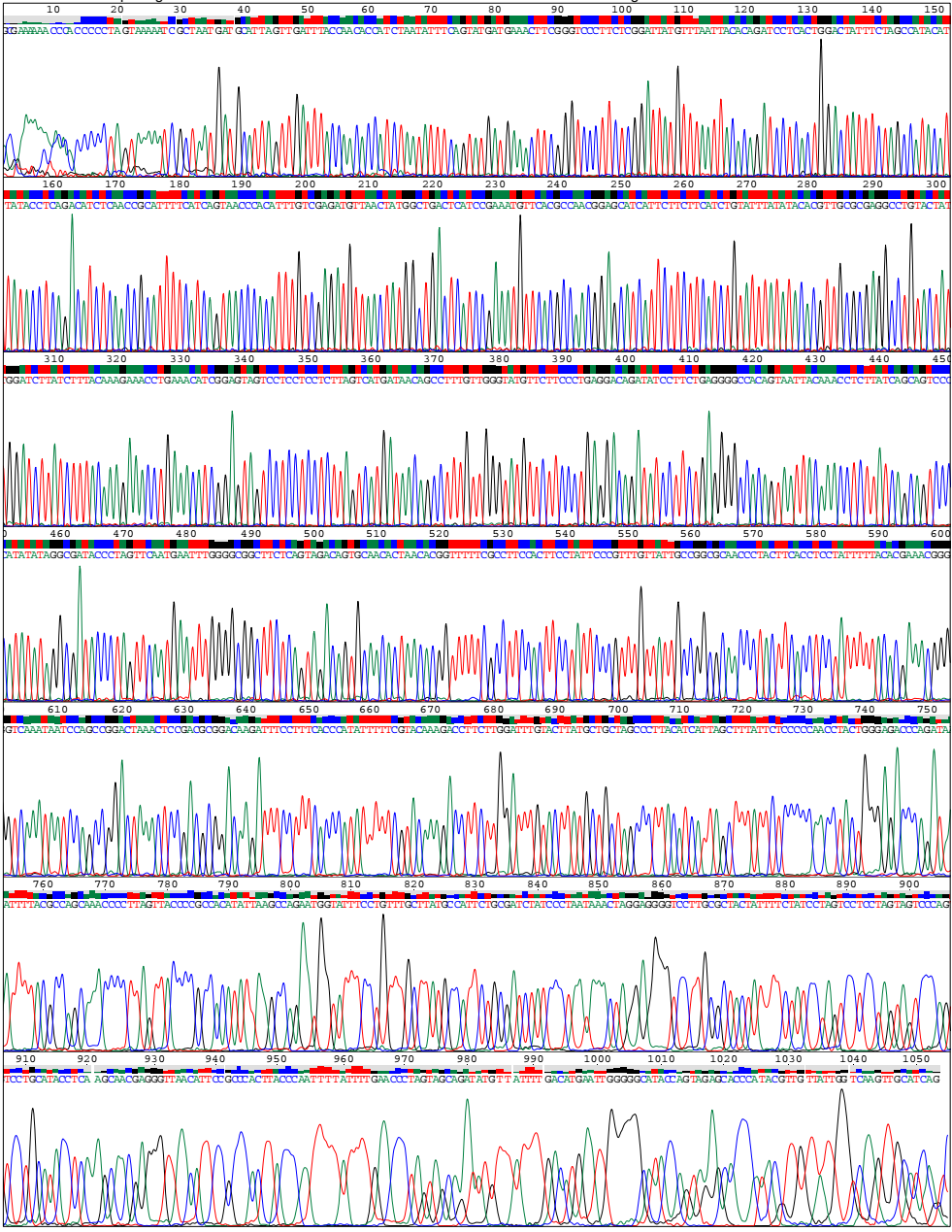


Fragment 12-posterior sequence (reverse sequencing)


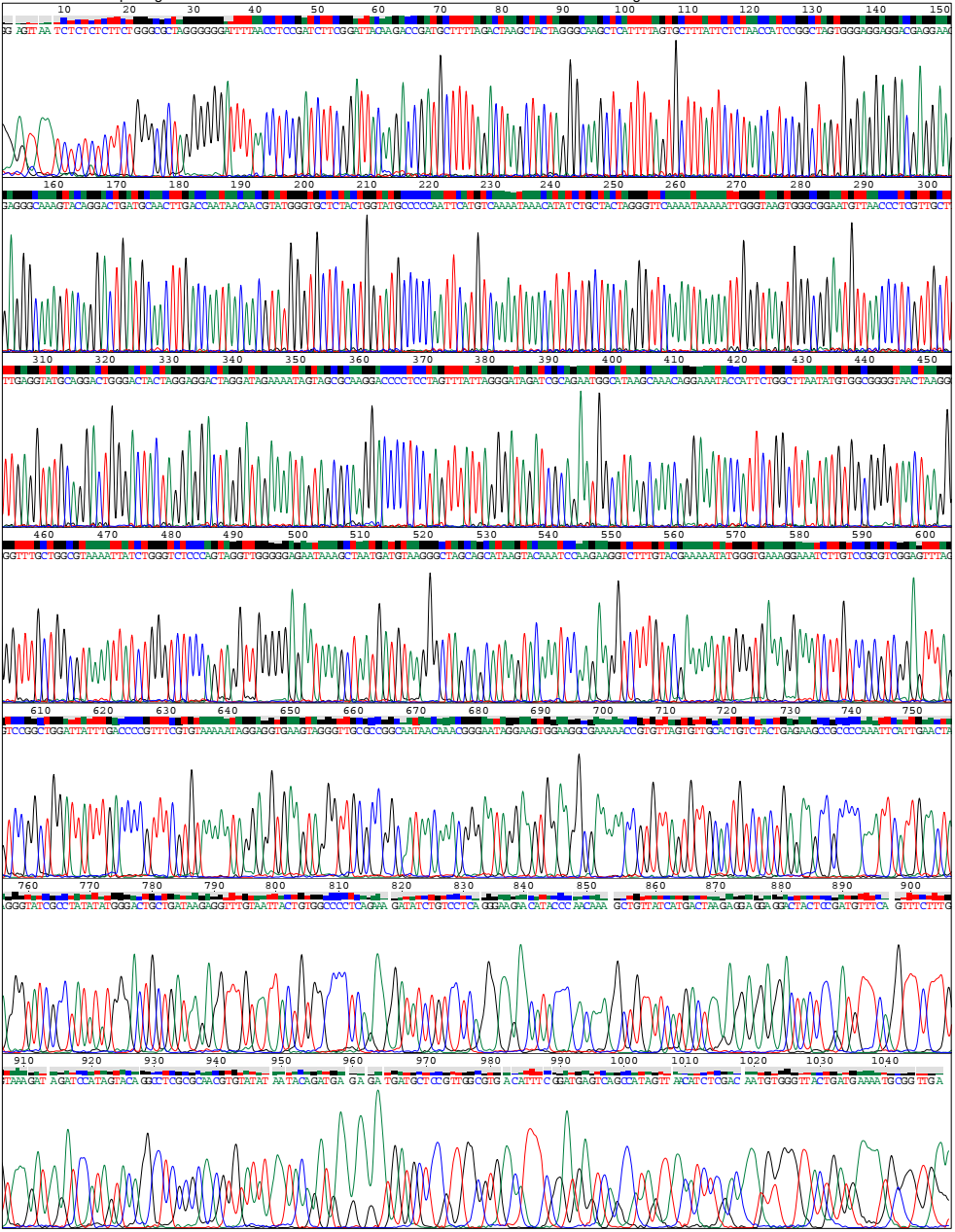


1. Fragment 13 was sequenced using primer pair 13.

Fragment 13-anterior sequence (forward sequencing)


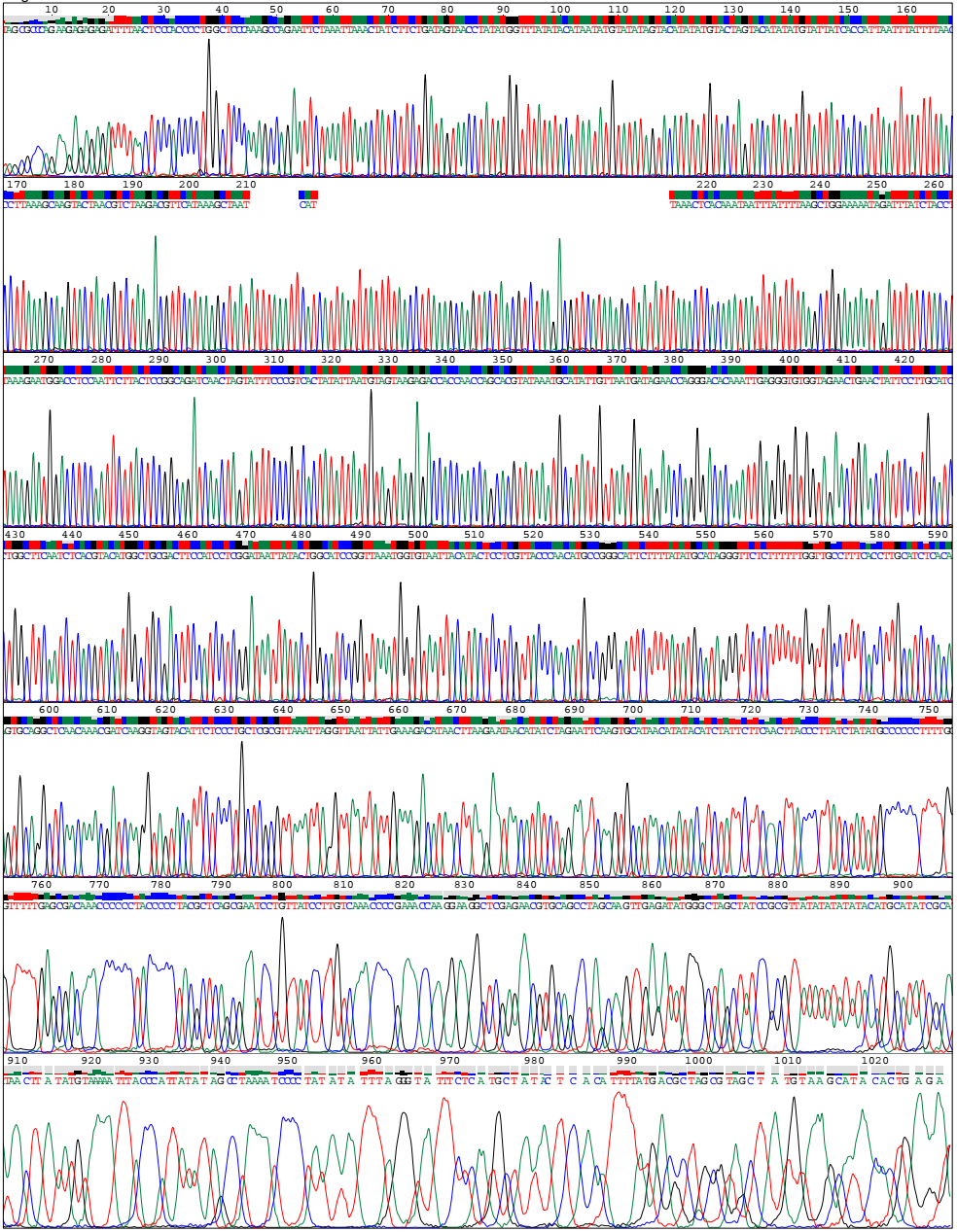


Fragment 13-posterior sequence (reverse sequencing)


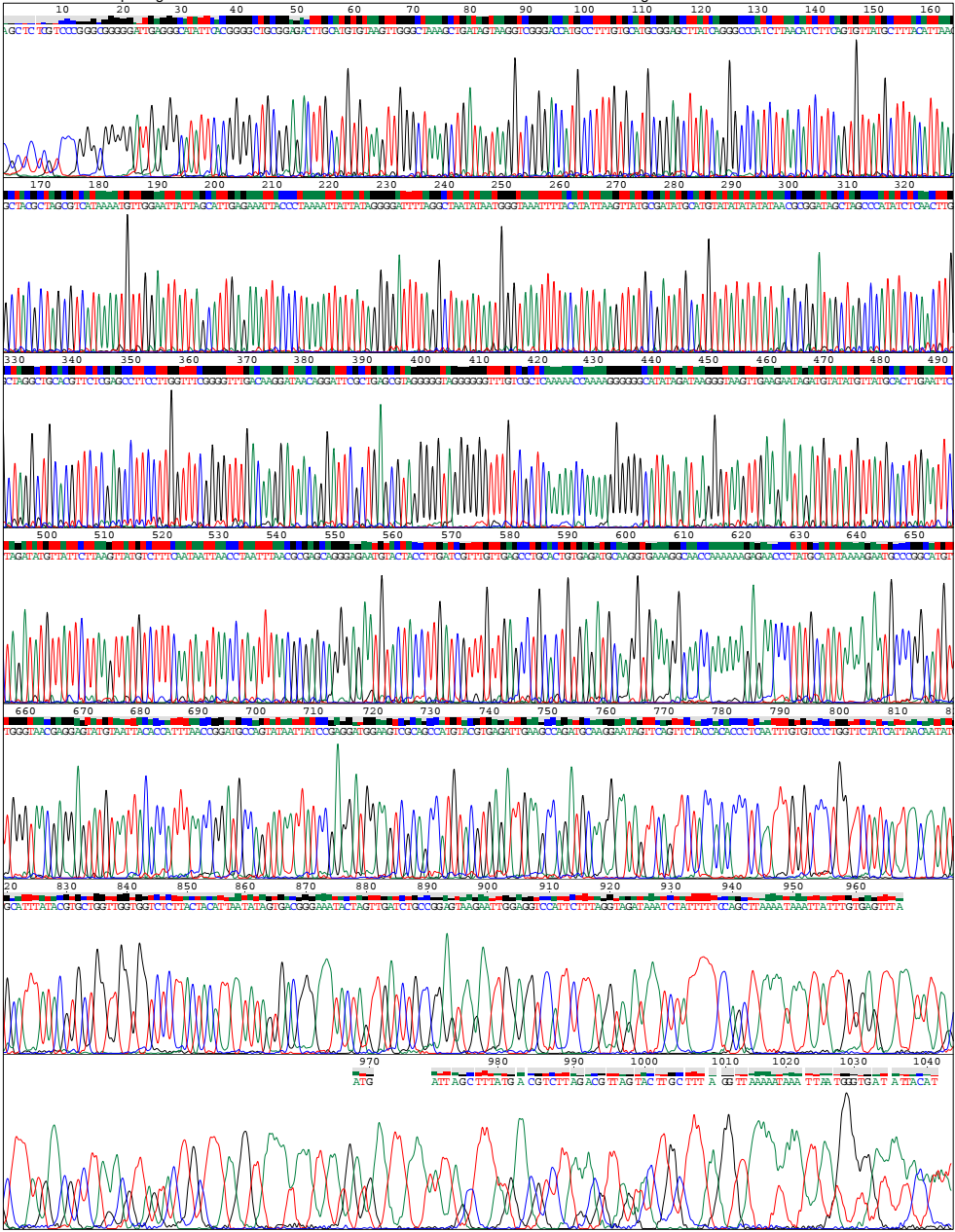

Supplement: Supplemental Material [file TMDN_A_2192310_SM6993.docx]
