# Supplementary material for: Complete mitochondrial genome of Belligobio pengxianensis (Cypriniformes: Gobionidae)
Source: Mitochondrial DNA B Resour. 2023 Mar 25;8(3):434–8. doi: 10.1080/23802359.2023.2192310 (PMC10044147; doi:10.1080/23802359.2023.2192310)
Supplement: Supplemental Material [file TMDN_A_2192310_SM6538.docx]

**Supplementary Material—Appendix I**

**Complete mitochondrial genome of *Belligobio pengxianensis* (Cypriniformes: Gobionidae)**

Bo Xuan, Mingyue Li, Xiaomin Ni and Cuizhang Fu*


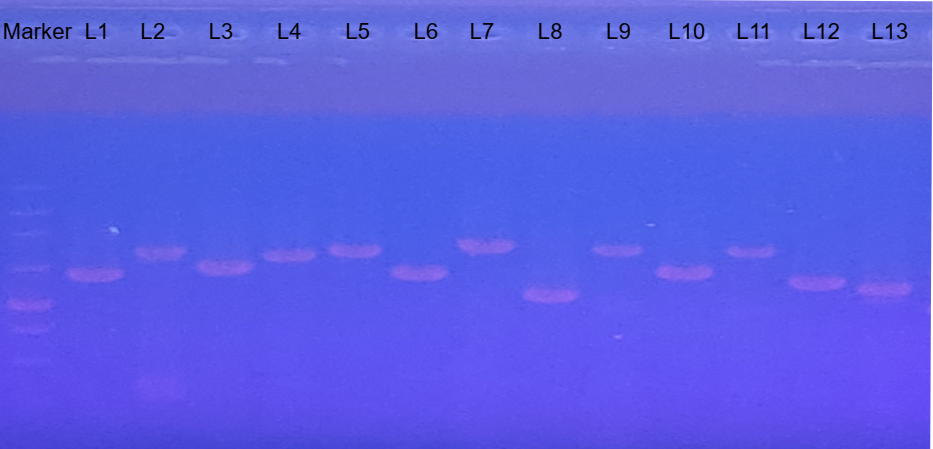


Figure S1 Gel electrophoresis map for mitochondrial PCR Products of *B. pengxianensis*. Shown are 250 bp DNA Ladder Marker and L1–13 represent 13 mitochondrial fragment amplified by primer pairs 1–13. Photographs by Bo Xuan on August 10, 2020.

Table S1 A total of 13 Primer pairs used in the PCR amplification of complete mitochondrial genome of *B. pengxianensis*

| Primer code | Primer name and sequence | Source | length |
| --- | --- | --- | --- |
| 1 | Gob12sF 5'-AAGGCATGGTCCYGACCTTA-3' | Chai and Fu, 2020 | 1404 bp |
|  | Gob16sR: 5'-TTCGGTAGGTCTRTCACTTC-3' | Chai and Fu, 2020 |  |
| 2 | Gob16sF: 5'-ACCTTGTACCTTTTGCATC-3' | Chai and Fu, 2020 | 1563 bp |
|  | GobLeuR: 5'-GGGAAGAGGAYTTGAACC-3' | Chai and Fu, 2020 |  |
| 3 | GobND1F: 5'-GCAGCCGCTATTAAGGGTT-3' | Chai and Fu, 2020 | 1501bp |
|  | GobND1R: 5'-GGRTTCATTGATGGAGGA-3' | Chai and Fu, 2020 |  |
| 4 | BPILeF: 5'-GGACCACTTTGATAGAG-3' | This study | 1588 bp |
|  | GobCOIR: 5'-CCAAATACRAGATARAGGT-3' | Chai and Fu, 2020 |  |
| 5 | GobAsnF: 5'-AGCGAGCATCCATCTACTT-3' | Chai and Fu, 2020 | 1794 bp |
|  | GobSerR: 5'-GGTYATGTGACTGGCTTGA-3' | Chai and Fu, 2020 |  |
| 6 | GobCOIF: 5'-TGAGAAGCCTTYGCCGCYAAACG-3' | Chai and Fu, 2020; | 1459 bp |
|  | BPATP8R: 5'-AGGAATACYATYAGGGAGGC-3' | This study |  |
| 7 | GobATP6F: 5'-CCTTGAGAYTGACCATGAT-3' | Chai and Fu, 2020 | 1943 bp |
|  | GobArgR: 5'-CTGAGYCGAAATCAGAGG-3' | Chai and Fu, 2020 |  |
| 8 | GobCOIIIF: 5'-TGATGAGGCTCATATCTTTCTA-3' | Chai and Fu, 2020 | 1139 bp |
|  | GobND4R: 5'-TCTGTGGCRCCRAATGCTAT-3' | Chai and Fu, 2020 |  |
| 9 | GobND4F: 5'-TAGCATTTCAYCGC ACMC-3' | Chai and Fu, 2020 | 1819bp |
|  | GobLeuR: 5'-TGGAYTTGCACCAAGAGT-3' | Chai and Fu, 2020 |  |
| 10 | GobSerF: 5'-ACTYACCRAGGAAGGACA-3' | Chai and Fu, 2020 | 1548 bp |
|  | GobND5R: 5'-TCCYCAGGCAAGYCGTTT-3' | Chai and Fu, 2020 |  |
| 11 | GobND5F: 5'-ATTGARGCCCTAAACACCTC-3' | Chai and Fu, 2020 | 1772 bp |
|  | GobCytbR: 5'-AAGTGGAAKGCGAARAATCG-3' | Chai and Fu, 2020 |  |
| 12 | BPGLU: 5'-AACCACCGTTGTAATTCAACTA-3' | This study | 1308 bp |
|  | BPROR:5'-TAGTTTAGTTTAGAATTCTGGCTTTGG-3' | This study |  |
| 13 | GobDloopF: 5'-AAAGCATCGGTCTTGTAATC-3' | Chai and Fu, 2020 | 1339 bp |
|  | GobDloopR: 5'-CTTGGCTAGGCGTCTTGG-3' | Chai and Fu, 2020 |  |
